# The pentafluorophenyl group as π-acceptor for anions: a case study†
†This manuscript is dedicated to Prof. Jean-Marie Lehn on the occasion of his 75th birthday.
‡
‡Electronic supplementary information (ESI) available. CCDC 967089, 967097, 1005267–1005289. For ESI and crystallographic data in CIF or other electronic format see DOI: 10.1039/c4sc02762k



**DOI:** 10.1039/c4sc02762k

**Published:** 2014-10-16

**Authors:** Michael Giese, Markus Albrecht, Arto Valkonen, Kari Rissanen

**Affiliations:** a Institut für Organische Chemie , RWTH Aachen University , Landoltweg 1 , 52074 Aachen , Germany . Email: markus.albrecht@oc.rwth-aachen.de; b Department of Chemistry , Nanoscience Center , University of Jyväskylä , Survontie 9 , 40014 Jyväskylä , Finland . Email: kari.t.rissanen@jyu.fi ; Fax: +358 14 260 2501 ; Tel: +358 50 562 3721

## Abstract

The present study gives a comprehensive insight into anion–π interactions in the solid state, focusing on purely organic and charge-neutral fluorophenyl groups bearing a positive charge located at a side chain. The detailed statistical analysis of a series of structural data sets shows the geometrical variability of anion–π bonding in the solid state. It reveals the directing substituents at the arene as key elements for the positional preferences of anions above π-systems. The structural variety of the interaction between anions and electron-deficient arenes is considered by use of the hapticity concept. Together with new evaluation criteria, two helpful tools to understand and describe anion–π interactions in the solid are used.

## Abstract 2

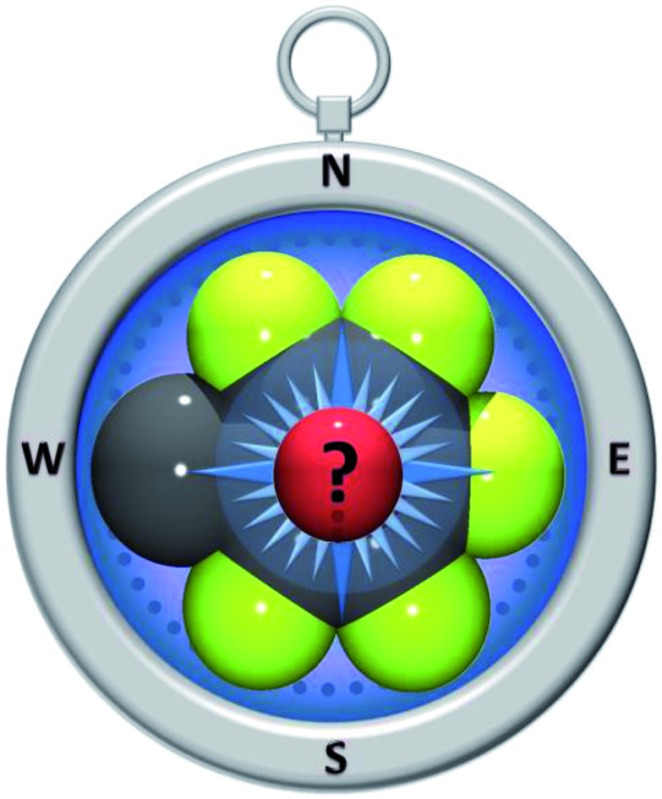
A unique structural study investigates the variability of anion–π bonding in the solid state structures of pentafluorophenyl arenes. The hapticity concept is used as tool to describe the structural differences of various anion–π complexes.

## Introduction

Supramolecular chemistry is defined as the chemistry of non-covalent interactions. Due to their broad versatility (from weak van der Waals interactions to hydrogen bonding and strong electrostatic attractions) they vary dramatically in their energetics.^1^ Often non-covalent interactions are weak compared to covalent bonds. However, due to cooperative and additive effects they are able to control molecular organization and assembly processes and form stable supramolecular aggregates. Another important feature of non-covalent interactions, reversibility and sensitivity to external stimuli provide the ability to correct errors in the assembly, self-heal structural disturbances, and create new functional materials.^2^

Only recently the interaction between anions and electron-deficient arenes has gained considerable attention, due to its potential application in anion sensing, recognition processes and relevance in enzyme activity.^3^ Since 2002 this intermolecular force is widely accepted as an influential attractive interaction.^3,4^ A series of computational as well as structural studies support the “existence” of anion–π interactions based on more or less arbitrary evaluation criteria.^3,5^ Thus, anion–π interactions were considered to be “commonplace”. In 2009 Hay and Custelcean reported an analysis of the orientation of anions towards π-systems using the Cambridge Structural Database (CSD).^6^ The strict criteria for anion–π interactions selected in this study evoked a controversial discussion, since it did not reveal a single example for such an interaction in the CSD.^7^

**Fig. 1:**
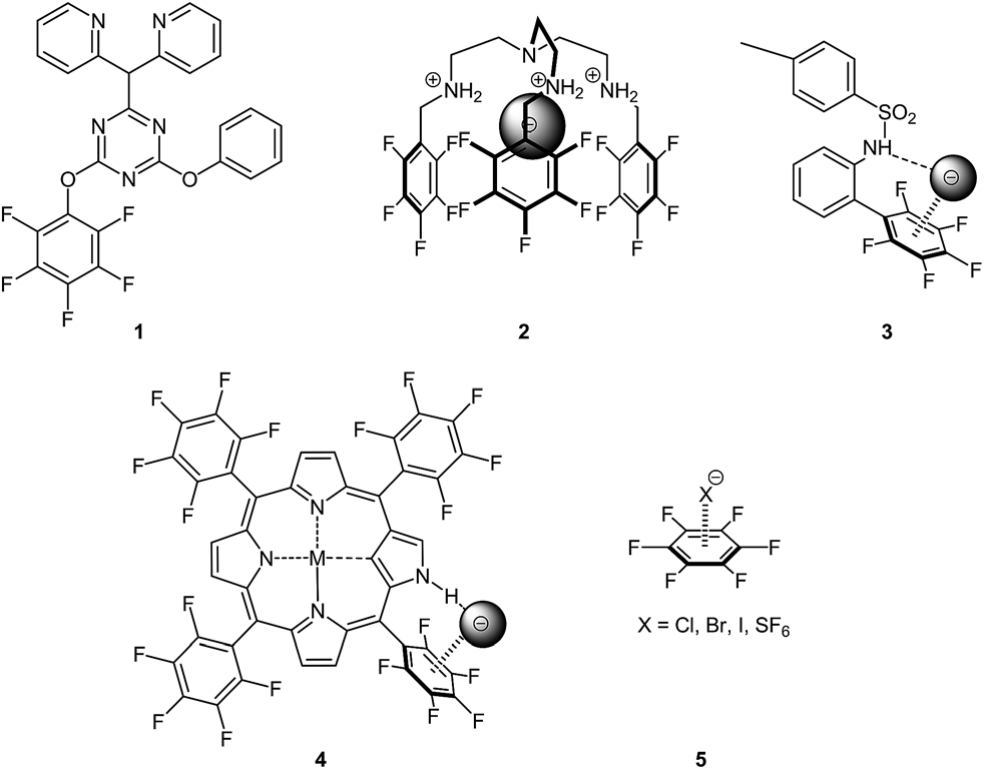
Pentafluorophenyl receptors of Reedijk (**1**),^9*a*^ Ghosh (**2**),^9b,c^ Johnson (**3**),^9*d*^ Maeda (**4**)^9e–g^ as well as Mizuse^9h,i^ and Weber (**5**)^9*j*^ for “anion–π interaction studies” in the crystal, solution or in the gas phase.

Since 2008, we investigate anion–π interactions of pentafluorophenyl derivatives.^8^ Due to the electron withdrawing nature of the fluorine atom this group shows an opposite charge distribution compared to corresponding phenyl derivatives. Only few examples by other groups were reported where this aromatic moiety was used for anion–π studies (see Fig. 1).^9^

In comparison to many previous investigations, a set of terms and conditions were chosen to systematically understand the interaction between anions and electron deficient arenes:

(1) The charge neutral pentafluorophenyl unit (C_6_F_5_) was chosen as electron deficient moiety in order to rule out that the interaction between the π-system and the anion is mainly based on electrostatic attraction which overrules a possible weak repulsion between the aromatic unit and the anion.

(2) A positive charge was established at the substituent of the C_6_F_5_ group either directly bound to the ring or separated by a spacer. This guarantees the presence of an anion in the vicinity of the electron deficient moiety.

## Results

**Fig. 2:**
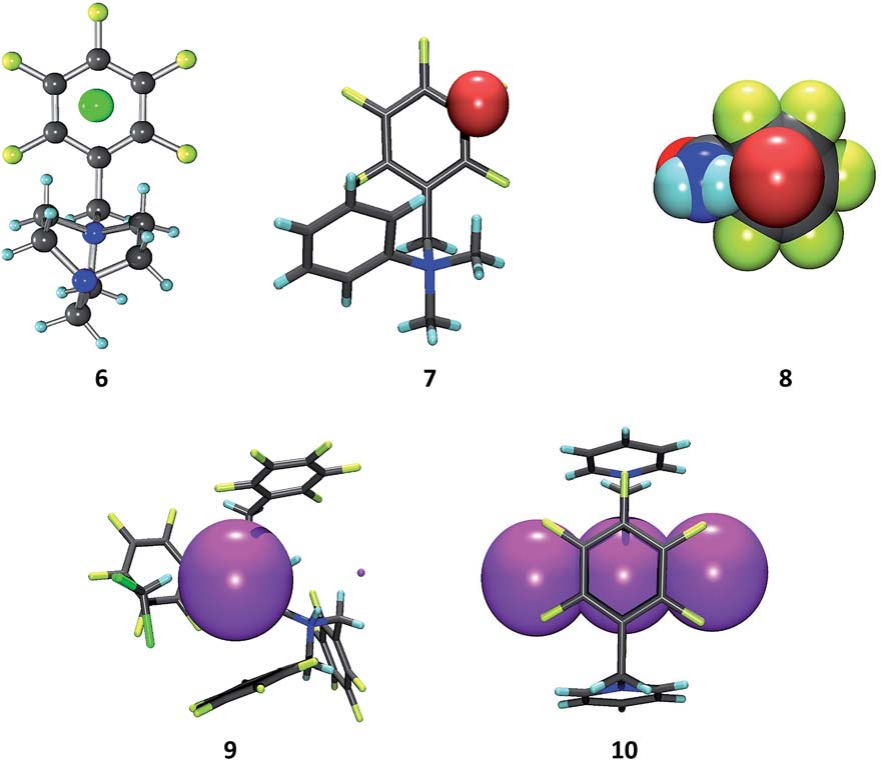
Representative structures of pentafluorophenyl receptors **6–10** in the crystal discussed in the present study showing the versatility of anion–π complexes as observed in the solid state (C: grey, F: yellow, Cl: green, Br: red, I: pink; co-crystallized solvent molecules and counter cations are omitted for clarity).^8b,e,f,i^

Our present study is in the way unique, as it analyses a huge data set of experimentally obtained structures of purely organic π-acceptor complexes of fluoroarenes (perfluorinated or partly fluorinated) with various anions. In contrast to previous reports, the focus is on the interaction of anions with uncharged π-systems. The role of the anion as well as of directing substituents in the periphery of the electron deficient arene is investigated. In this work, the positions of 101 anions obtained are systematically compared from 80 crystal structures determined by us, 25 in this work (see ESI‡), the remaining 55 being published earlier,^8^ with the aim to get a comprehensive picture of the versatility of anion–π contacts in the solid state (see Fig. 2).

### Criteria to describe interactions between anions and π-systems

In order to get an overview of the investigated structures, the positions of 101 anions (8 chloride, 58 bromide, 17 iodide and 17 structures with other anions such as tetrafluoroborate, hexafluorophosphate or nitrate) were plotted in a normalized coordinate system. Thereby, the fluorinated phenyl group is located in the *xz*-plane with its centre in the zero point.

**Fig. 3:**
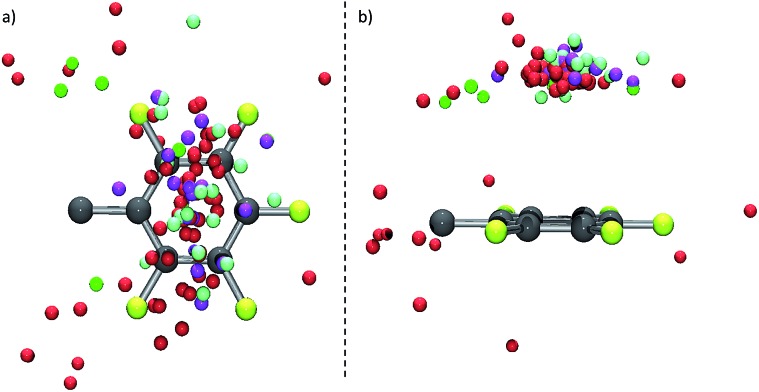
Graphical analysis of 101 anion positions in respect to a normalized (penta)fluoroarene. (A) Top view; (B) side view (C: grey, F: yellow, Cl: green, Br: red, I: pink, other anions (*e.g.* BF_4_^–^, PF_6_^–^, NO_3_^–^, I_3_^–^, BrIBr^–^, I_4_^2–^): light green).

The graphical analysis (Fig. 3) shows that the majority of the anions (91, 89%) are located above (or below) the plane of the arene (on-top position). Only 11 anions are found in the outer periphery of the arene (side-on position). In those examples, either cocrystallized solvent molecules force the anions away from the top of the π-system or they are bound to *ortho*-hydrogen atoms of partially fluorinated arenes (few examples of C_6_HF_4_, C_6_H_2_F_3_, C_6_H_3_F_2_ derivatives are included in here).^8*e*^ Anions located on-top of the π-system show a broad positional distribution above the plane of the arene with a focus of the density of the anions near the centre of the aromatic unit.

As mentioned in the introduction, anion–π interactions in the solid state are regarded as either “commonplace or extraordinary” depending on the selected evaluation criteria. In here different reported criteria were applied on the collected structural data set. The early criteria of Deyà *et al.* (2002: distance between arene-carbon atoms and anion <∑vdW + 1.0 Å)^5*b*^ and Reedijk and coworkers (2008: distance between centre of arene, any of the arene atoms and the anion <∑4.0 Å)^10^ lead to 54 or 40 hits, respectively, for halide containing anion–π aggregates. More restricted evaluation criteria by Hay and Custelcean (2009: distance between each arene-carbon atom and anion <∑vdW + 0.2 Å)^6^ do not lead to any anion–π contact in the analysis of the pentafluorophenyl system. In 2011 Deyà and coworkers reported modified criteria for anion–π interactions considering the structural variability of the relative position of the anion above the π-system (distance between arene-carbon atoms and anion <∑vdW + 0.8 Å).^7^ This leads to 36 hits of anion–π complexes within the present study.

Since crystal structures are the result of a complex interplay of non-covalent interactions as packing effects anions might be forced in unfavourable positions in the crystal lattice. Based on this, the initial 2002 criteria of Deyà seem to be too loosely selected. On the other hand, anion–π interactions easily can be influenced by other non-covalent interactions. Due to directing effects of the substituents at the fluoroarene the position above the centre of the π-system does not have to be the most favourable one for the anion. Therefore, Hay's 2009 criteria would be correct, if the interaction would have a high covalent contribution. However, anion–π interactions are mainly based on non-directed electrostatic attraction. Consequently, the interaction of molecular species with a π-system should not be restricted to the position directly above the centre of an arene and the exact sum of van der Waals radii is not a sufficient criterion to evaluate anion–π contacts. For example, cation–π interactions of phenyl groups can span the whole range from η^1^ to η^6^. Based on this, a definition for anion–π interactions using the “hapticity concept” in combination with newly adjusted criteria are recommended in here:

(1) The anion has to be located above the π-system. Therefore, any centroid-ring atom–anion angle should not be higher than 90° (+10% of tolerance).

(2) The distance between the anion and the plane of the π-system has to be shorter than ∑vdW + 0.4 Å (the problem of a fixed number for the vdW radii of halogen anions is discussed in ref. 7).

(3) The hapticity of the anion–π interactions is given by the number of contacts to ring atoms which are <∑vdW + 0.4 Å.

The main text of the article should go here with headings as appropriate.

An alternative to report the hapticity would be to list the “offset” of the anion from the center of the aromatic unit in order to describe the relative position of the anion to the aromatic ring, which is easy to quantify. However, to us the simple hapticity seems to be more descriptive and easier comparable.

The evaluation criteria proposed in here are based on our experience with solid state anion–π interactions. For an attractive interaction between an anion and an electron-deficient arene the anion has to be located on top of the arene (criterion 1) in close proximity to the arene plane (criterion 2). The 0.4 Å which are added to the vdW radii are due to a recent adjustment of the tabulated vdW radii of halide anions as reported by Deyà and coworkers in 2011.^7^ The final criterion introduces a tool to describe the versatility of anion–π interactions in the solid state by a simple consideration of the given arene atom···anion distances.

### Selected crystal structures

**Fig. 4:**
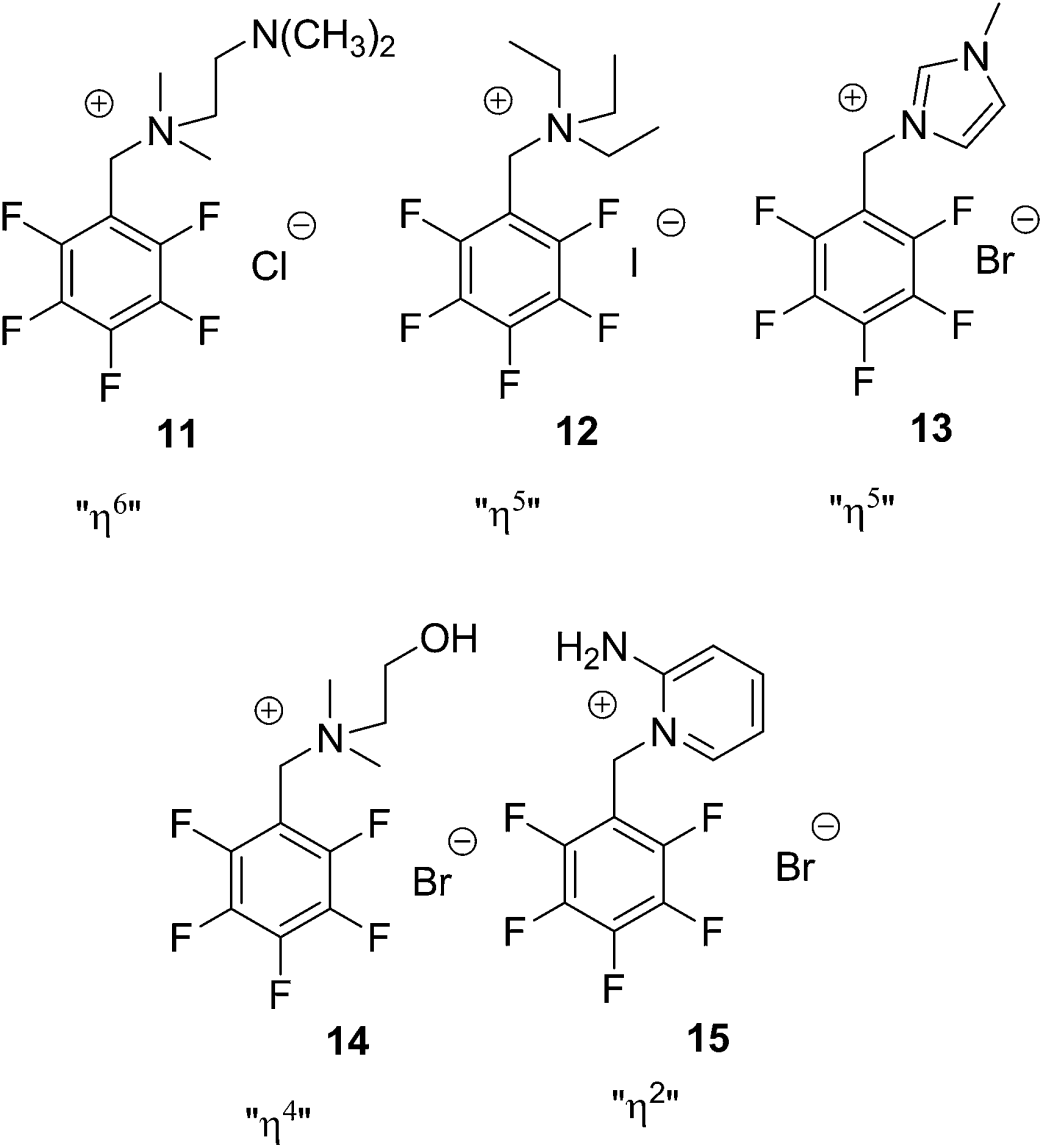
Selected examples of pentafluorophenyl derivatives with different cationic side chains.

The defined criteria will be illustrated by the crystal structures of the chloride **11**, the iodide **12** as well as the bromide salts **13–15** represented in Fig. 4. The derivatives bear different cationic units (ammonium *versus* imidazolium *versus* aminopyridinium) in the side chain. In the discussion of the crystal structures only relevant distances and interactions will be described although there are many more present.

**Fig. 5:**
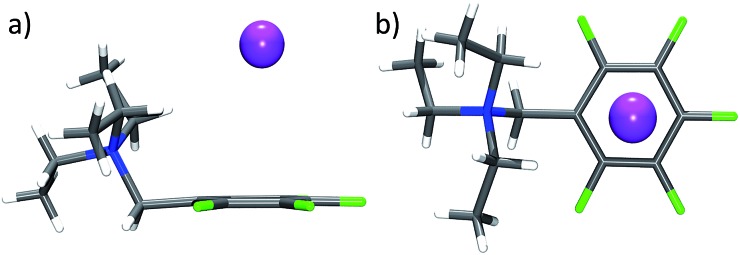
Structure of **11** in the crystal exhibiting the η^6^ interaction between the pentafluorophenyl group and chloride. The co-crystallized water was omitted for clarity.

The crystal structure of **11** represents the ideal case of anion–π contacts (see Fig. 5). The chloride is fixed directly on top of the centre of the pentafluorophenyl unit by two non-classical hydrogen bonds of the two methyl groups (CH···Cl = 2.92, 2.94 Å). All six carbon···anion distances match our criteria (C1–C6 = 3.50–3.83 Å) resulting in the “η^6^”-type interaction. However, the shortest non-covalent contacts in the solid state structure of **11** are observed between the anion and the cocrystallized water (HOH···Cl = 2.43 Å).

**Fig. 6:**
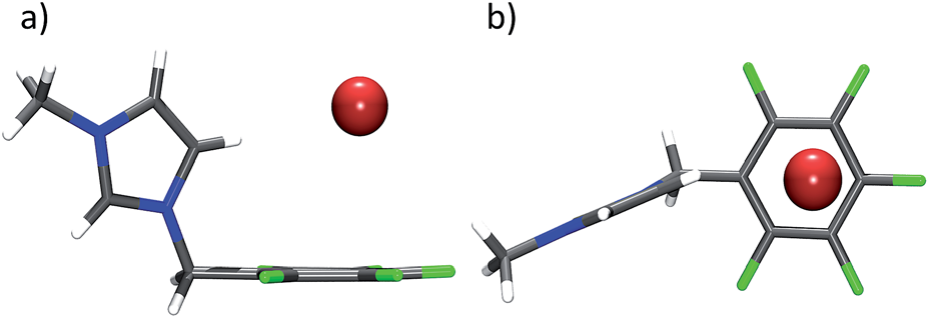
Structure of **12** in the crystal exhibiting the η^5^ interaction between the pentafluorophenyl group and iodide.

The closely related structure of compound **12** is shown in Fig. 6. Iodide is located close to the cationic ammonium group as well as to the pentafluorophenyl π-acceptor. Interaction with the tetraethylammonium unit takes place by non-classical hydrogen bonds between iodide and α-CH of two ethyl groups (CH···I = 3.17, 3.22 Å). The iodide is located close to the center of the pentafluorophenyl unit with distances to carbon atoms of 4.10 (C1), 4.07/4.05 (C2/C6), 3.98/3.99 (C3/C5) and 3.94 Å (C4). The distance between iodide and C1 slightly exceeds our limit resulting in a “η^5^” type arrangement. However, due to the bulkiness of the ammonium substituent, the anion is somewhat shifted from the center towards C4 prohibiting “η^6^”.

**Fig. 7:**
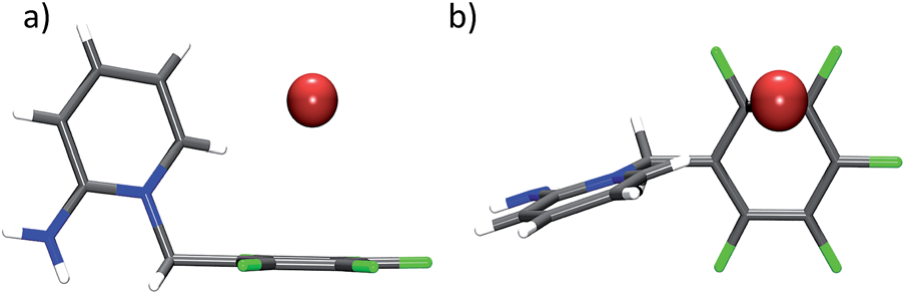
Structure of **13** in the crystal exhibiting the η^5^ interaction between the pentafluorophenyl group and bromide.

The relevant anion cation pair of **13** as found in the crystal is depicted in Fig. 7. The imidazoline plane is somewhat tilted against the C1–C4 axis of the fluorinated phenyl group with the CH=CH unit pointing towards the electron deficient ring. Imidazoline-CH in 5-position binds to the bromide anion (H···Br = 2.77 Å). Distances between bromide and the ring carbon atoms are 4.04 (C1), 3.91 (C2), 3.65 (C3), 3.46 (C4), 3.59 (C5) and 3.87 Å (C6). The interaction to C1 clearly exceeds our limit and the structure again can be described to be η^5^.

**Fig. 8:**
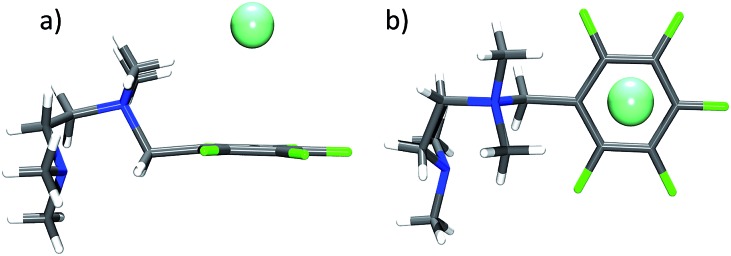
Solid state structure of **14** illustrating the η^4^-type arrangement of the bromide relative to the pentafluorophenyl unit.

In order to show the versatility of observed anion–π contacts in the present solid state study we depict in addition the crystal structure of **14** (Fig. 8). Similar to the structures of **11** and **12** the bromide is fixed by non-classical hydrogen bonds of the alkyl groups close to the pentafluorophenyl system (CH···Br = 3.41, 3.71 Å). However, in contrast to the structures described above, the anion is shifted towards the rim of the π-system leading to four C···Br distances within the applied evaluation criteria (C3–C6 = 3.92, 3.54, 3.43 and 3.70 Å) resulting in a “η^4^”-type arrangement of the anion relative to the electron-deficient arene.

**Fig. 9:**
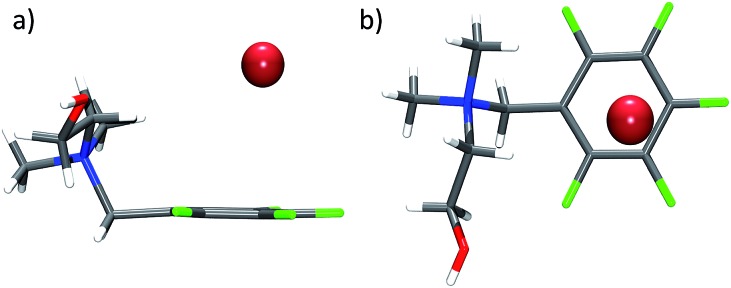
Structure of **15** in the crystal exhibiting the η^2^ interaction between the pentafluorophenyl group and bromide.

Structure **15** is very different (Fig. 9). The 2-aminopyridin substituent is orientated with the amino group away from the pentafluorophenyl moiety. The amine undergoes hydrogen bonds to bromide anions which are not relevant for the present discussion. The interaction of H6 of the pyridine to bromide (2.77 Å) is relevant. It directs the anion over the aromatic unit, not at the centre but at the rim. Carbon bromide distances are 4.33 (C1), 3.69 (C2), 3.60 (C3), 4.15 (C4), 4.73 (C5), and 4.80 Å showing clearly the η^2^ mode of interaction.

### Interactions of halides with pentafluorophenyl derivatives

Due to the spherical shape and easy determination of their position, halides are ideal anions to analyze anion–π interactions. Therefore the following discussion will focus on this class of anions.

Applying those criteria to our data set leads to 48 anion–π contacts for the halide structures containing pentafluorophenyl unit. A summary of the structural findings depending on the applied search criteria is given in Table 1.

**Table 1:** Anion–π contacts of investigated halide derivatives in respect to the applied evaluation criteria

	Reedijk *et al.* 2008 (ref. 10)	Hay *et al.* 2009 (ref. 6)	Deyà *et al.* 2002 (ref. 5*b*)	Deyà *et al.*2011 (ref. 7)	This study 2014
Cl	3	0	3	3	6
Br	33	0	35	25	29
I	11	0	16	8	13
∑	47	0	54	36	48

**Fig. 10:**
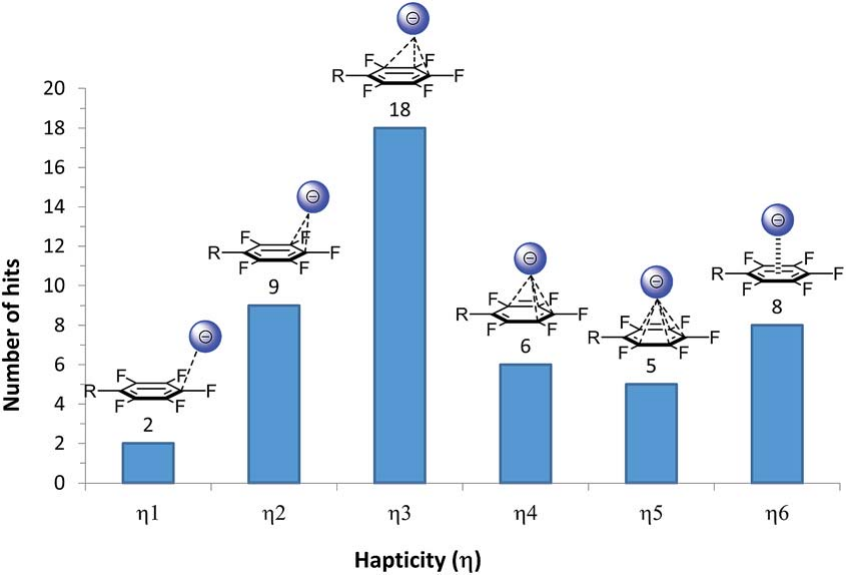
Structural analysis of the halides located above the electron-deficient π-systems according to the binding motif.

In order to evaluate the position of the anion in respect to the π-system, the “hapticity” (the number of reasonable bonding contacts between an anion and carbon atoms of the arene) was determined (Fig. 10). In eight cases the anions are located close to the centre of the arene and matching the criteria for all six carbon atoms, thus they show a η^6^-type binding motif. However, the majority of anions (40 anions) interacts partially with the electron-deficient π-system (η^1^–η^5^ binding) and, interestingly, in only two structures the anion is located close to only one carbon atom of the pentafluorophenyl unit.

**Fig. 11:**
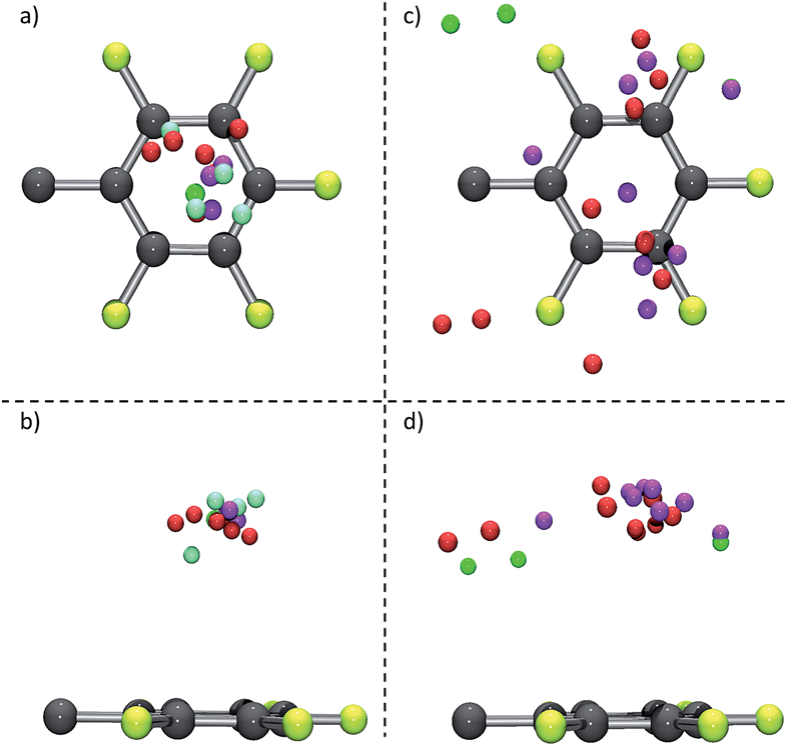
Comparative, graphical analysis for structures of π-acceptors with a symmetrical, rigid scaffold and without cocrystallized solvent molecules; (a) top view; (b) side view, as well as for more flexible and less symmetric scaffolded acceptor systems; (c) top view and (d) side view (C: grey, F: yellow, Cl: green, Br: red, I: pink, other anions (BF_4_^–^ and PF_6_^–^ are included in addition to the halides): light green).

The structural comparison of the crystallographic data reveals a significant influence of the scaffold formed by the substituent of the π-acceptors. Those structures where the side group of the π-acceptor (i) is rigid, (ii) does not introduce dissymmetry and (iii) any disturbing cocrystallized solvent molecules are absent (see **6**, Fig. 2 as a representative example for an example) lead to fourteen hits within our collection of data with anions located close above the centre of the electron-deficient arene (see Fig. 11a and b). In contrast to that, corresponding structures with more flexible and less symmetric scaffolds (see **7**, Fig. 2 as a representative example) show a significantly broader distribution of the anion position above the ring (see Fig. 11c and d for an example). This observation supports our previous suggestion that the position of the anion in respect to the π-acidic unit strongly depends on the substituents in the periphery of the arene and can be controlled by those.^8a,b^

## Conclusions

In conclusion, the presented statistical approached based on systematically obtained experimental data, investigates metal-free and charge-neutral fluorophenyl derivatives in respect to their π-acceptor ability for anions. Positive charge is only introduced in the periphery of the arene side chains. The results clearly show that anions prefer the position above the electron-deficient π-system (nearly 90%). The high variability of the anion position is caused by interplay of various non-covalent interactions. Thereby, directing substituents at the fluoroarene play a key role. The introduced concept of hapticity as well as the proposed evaluation criteria are helpful to sufficiently describe anion–π interactions in the crystal.
