# Supplementary material for: The pentafluorophenyl group as π-acceptor for anions: a case study
Source: Chem Sci. 2014 Oct 16;6(1):354–9. doi: 10.1039/c4sc02762k (PMC5586208; doi:10.1039/c4sc02762k)
Supplement: Supplementary file 1 [file SC-006-C4SC02762K-s001.pdf]

Electronic Supplementary Material (ESI) for Chemical Science.
This journal is © The Royal Society of Chemistry 2014

Supporting Information for

The pentafluorophenyl group as π-acceptor for Anions: A case study

Michael Giese, Markus Albrecht*, Arto Valkonen and Kari Rissanen*

I. Synthetic Procedures and Characterization of Compounds

General

All commercially available reagents were used as received. Solvents were distilled before use. The obtained compounds were fully characterized by standard analytical methods using a Varian Mercury 300 NMR spectrometer for ^{1}H- (300 MHz) and ^{19}F-NMR spactra (282 MHz) in deuteric solvents. Mass spectrometric data were recorded on Finnigan SSQ 7000 and Thermo Deca XP as EI (70 eV) or ESI. IR spectra were obtained by a PerkinElmer FTIR spectrometer Spektrum 100 in KBr pellets (4000-650 cm^{-1}) and a CHN-O-Rapid Vario EL from Heraeus was used for elemental analysis. Melting points were taken by a Büchi B-540 without correction.

Synthesis and Characterisation of Compounds 1-20

Triethyl-fluorobenzylammonium Salts:

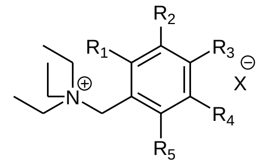

1: R_1, R_2, R_4, R_5 = F; R_3 = H; X = Br^-
2: R_1, R_2, R_3, R_4 = F; R_5 = H; X = Br^-
3: R_1, R_3, R_5 = F; R_2, R_4 = H; X = Br^-
4: R_2, R_3, R_4 = F; R_1, R_5 = H; X = Br^-
5: R_2, R_4 = F; R_1; R_3, R_5 = H; X = Br^-
6: R_1-R_5 = F; X = I^-

The triethyl-fluorobenzylammonium salts were synthesized following the general procedure. In a typical procedure 200 mg of triethylamine (1.0 eq., 1.98 mmol) were dissolved in 20 mL of chloroform. Then the corresponding amount of the fluorobenzyl halide (1.0 eq.) dissolved in 10 mL of chloroform was added and the reactions mixture was stirred for 12 h at room temperature. The solvent was removed under reduced pressure and the remaining solid was dried in vacuo.

2,3,5,6-Tetrafluorobenzyl-triethylammonium bromide (1)
230 mg colorless solid (M = 343.04 g/mol, 0.70 mmol, 85 %); **mp:** 249 °C
^{1}H-NMR (300 MHz, MeOD): δ (ppm) = 7.83-7.71 (m, 1H, H_{aryl}), 4.73 (s, 2H, H_{benzyl}), 3.44 (q, J = 7.2 Hz, 6H, CH_2), 1.45 (t, J = 7.2 Hz, 9H, CH_3).
^{19}F-NMR (300 MHz, MeOD): δ (ppm) = -138.18 (m, 2F, F_{ortho}), -139.01 (m, 2F, F_{meta}).
MS (ESI): m/z (%) = positive: 264.8 (12, $[M]^+$, $C_{13}H_{18}NF_4^+$), 607.7 (100, $[M_2Br]^+$, $C_{23}H_{36}N_2F_8Br^+$).

IR (KBr): ν (cm^{-1}) = 2969 (m), 1804 (w), 2677 (w), 1810 (w), 1749 (w), 1617 (w), 1507 (vs), 1457 (m), 1398 (m), 1378 (m), 1316 (m), 1259 (s), 1191 (s), 1159 (m), 1112 (w), 1031 (m), 1008 (vs), 940 (m), 902 (s), 846 (m), 815 (m), 799 (m), 749 (w), 713 (s), 680 (w), 658 (m).
Elemental Analysis (%): calculated $C_{13}H_{18}F_4NBr$: C 45.36, H 5.27, N 4.07; found: C 45.08, H 5.37, N 4.09.
Two sets of colorless crystals from DMF/Et_2O (see Table S2 and Table S3).

2,3,4,5-Tetrafluorobenzyl-triethylammonium bromide (2)
290 mg colorless solid (M = 343.04 g/mol, 0.80 mmol, 99%); **mp:** 282 °C
^{1}H-NMR (400 MHz, MeOD): δ (ppm) = 7.54-7.47 (m, 1H, H_{aryl}), 4.60 (s, 2H, H_{benzyl}), 3.35 (q, J = 7.2 Hz, 6H, CH_2), 1.41 (t, J = 7.2 Hz, 9H, CH_3).
^{19}F-NMR (400 MHz, MeOD): δ (ppm) = -137.29 (m, 1F), -139.67 (m, 1F), -153.54 (m, 1F), -156.36 (m, 1F).
MS (ESI): m/z (%) = positive: 264.33 (18, $[M]^+$, $C_{13}H_{18}NF_4^+$), 607.67 (100, $[M_2Br]^+$, $C_{23}H_{36}N_2F_8Br^+$).
IR (KBr): ν (cm^{-1}) = 3465 (w), 2986 (m), 2758 (w), 2673 (w), 2487 (w), 2080 (w), 1991 (w), 1839 (w), 1633 (m), 1527 (vs), 1493 (vs), 1405 (m), 1365 (s), 1311 (m), 1270 (m), 1224 (m), 1179 (m), 1117 (vs), 1058 (vs), 1025 (vs), 946 (vs), 895 (s), 798 (vs), 716 (s), 664 (m).
Elemental Analysis (%): calculated $C_{13}H_{18}F_4NBr$: C 45.36, H 5.27, N 4.07; found: C 45.16, H 5.29, N 4.04.
Colorless crystals from DMF/Et_2O (see Table S4).

2,4,6-Trifluorobenzyl-triethylammonium bromide (3)
350 mg colorless solid (M = 325.07 g/mol, 0.77 mmol, 86%); **mp:** 215 °C
^{1}H-NMR (300 MHz, MeOD): δ (ppm) = 7.14 (t, J = 8.9 Hz, 2H, H_{aryl}), 4.58 (s, 2H, H_{benzyl}), 3.38 (q, J = 7.2 Hz, 6H, CH_2), 1.42 (t, J = 7.2 Hz, 9H, CH_3).
^{19}F-NMR (300 MHz, MeOD): δ (ppm) = -103.18 (m, 1F, F_{para}), -106.25 (m, 2F, F_{ortho}).
MS (ESI): m/z (%) = positive: 246.7 (12, $[M]^+$, $C_{13}H_{19}F_3N^+$), 571.7 (100, $[M_2Br]^+$, $C_{26}H_{38}F_6N_2Br^+$).
IR (KBr): ν (cm^{-1}) = 2997 (m), 2967 (m), 2678 (w), 2086 (w), 1740 (m), 1671 (w), 1630 (s), 1600 (s), 1470 (s), 1443 (s), 1417 (w), 1399 (m), 1374 (m), 1337 (w), 1316 (w), 1272 (m), 1211 (m), 1187 (m), 1160 (m), 1122 (vs), 1054 (s), 1020 (s), 995 (s), 939 (m), 886 (vs), 806 (s), 791 (s), 731 (m), 670 (w).
Elemental Analysis (%): calculated $C_{13}H_{19}F_3NBr$: C 47.87, H 5.87, N 4.29; found: C 48.08, H 6.02, N 4.35.
Colorless crystals from DMF/EE (see Table S5).

3,4,5-Trifluorobenzyl-triethylammonium bromide (4)
270 mg colorless solid (M = 325.07 g/mol, 0.83 mmol, 93%); **mp:** 227 °C
^{1}H-NMR (300 MHz, MeOD): δ (ppm) = 7.42 (t, J = 7.5 Hz, 2H, H_{aryl}), 4.50 (s, 2H, H_{benzyl}), 3.36-3.28 (m, 6H, CH_2), 1.42 (t, J = 7.3 Hz, 9H, CH_3).
^{19}F-NMR (300 MHz, MeOD): δ (ppm) = -134.96 (m, 2F, F_{meta}), -159.97 (m, 1F, F_{para}).
MS (ESI): m/z (%) = positive: 246.5 (15, $[M]^+$, $C_{13}H_{19}F_3N^+$), 571.4 (100, $[M_2Br]^+$, $C_{26}H_{38}F_6N_2Br^+$).
IR (KBr): ν (cm^{-1}) = 3486 (w), 3418 (w), 2977 (m), 2038 (w), 1737 (w), 1614 (m), 1531 (vs), 1453 (s), 1397 (m), 1352 (s), 1277 (w), 1237 (m), 1180 (m), 1150 (m), 1045 (vs), 939 (w), 860 (m), 789 (vs), 709 (m), 669 (m).
Elemental Analysis (%): calculated $C_{13}H_{19}F_3NBr$: C 47.87, H 5.87, N 4.29; found: C 47.30, H 5.73, N 4.35.
Colorless crystals from DMF/EE (see Table S6).

3,5-Difluorobenzyl-triethylammonium bromide (5)
265 mg colorless solid (M = 307.07 g/mol, 0.80 mmol, 89%); **mp:** 241 °C
^{1}H-NMR (300 MHz, MeOD): δ (ppm) = 7.26-7.20 (m, 3H, H_{aryl}), 4.52 (s, 2H, H_{benzyl}), 3.37-3.27 (m, 6H, CH_2), 1.42 (t, J = 7.3 Hz, 9H, CH_3).
^{19}F-NMR (300 MHz, MeOD): δ (ppm) = -109.50 (m, 2F, F_{meta}).
MS (ESI): m/z (%) = positive: 228.4 (12, $[M]^+$, $C_{13}H_{20}F_2N^+$), 535.5 (100, $[M_2Br]^+$, $C_{26}H_{40}F_4N_2Br^+$).
IR (KBr): ν (cm^{-1}) = 3006 (s), 2762 (w), 1797 (w), 1620 (s), 1596 (vs), 1517 (w), 1454 (vs), 1403 (m), 1367 (w), 1314 (vs), 1283 (m), 1232 (w), 1177 (m), 1130 (vs), 967 (s), 900 (s), 867 (s), 800 (vs), 719 (m), 661 (m).
Elemental Analysis (%): calculated $C_{13}H_{20}F_2NBr$: C 50.66, H 6.54, N 4.54; found: C 50.49, H 6.39, N 4.53.
Colorless crystals from DMF/EE (see Table S7).

Pentafluorobenzyl-triethylammonium iodide (6)
300 mg colorless solid (M = 409.03 g/mol, 0.73 mmol, 92%); **mp:** 176 °C
^{1}H-NMR (300 MHz, $CDCl_3$): δ (ppm) = 4.85 (s, 2H, H_{benzyl}), 3.60 (q, J = 7.2 Hz, 6H, CH_2), 1.52 (t, J = 7.2 , 9H, CH_3).
^{19}F-NMR (300 MHz, $CDCl_3$): δ (ppm) = -134.87 (m, 2F, F_{ortho}), -146.04 (m, 1F, F_{para}), -158.02 (m, 2F, F_{meta}).
MS (ESI): m/z (%) = positive: 282.25 (100, $[M]^+$, $C_{13}H_{17}F_5N^+$).
IR (KBr): ν (cm^{-1}) = 2968 (w), 2761 (w), 2675 (w), 2179 (w), 2105 (w, brd), 1991 (w), 1953 (w), 1747 (w), 1657 (m), 1521 (vs), 1504 (vs), 1476 (s), 1455 (s), 1402 (s), 1381 (m), 1308 (m), 1261 (w), 1217 (w), 1160 (m), 1131 (s), 1074 (w), 1016 (vs), 979 (s), 897 (w), 862 (w), 796 (s), 744 (w), 731 (w), 685 (w), 669 (m).
Elemental Analysis (%): calculated $C_{13}H_{17}NF_5I$: C 38.16, H 4.19, N 3.42; found: C 37.46, H 3.92, N 3.27.
Colorless crystals from DMF/Et_2O (see Table S8).

Dimethylbenzyl-fluorobenzylammonium Salts:

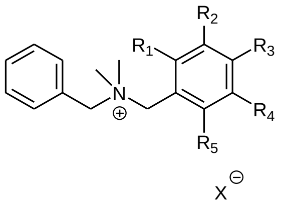

7: R_1-R_5 = F; X = Br^-
8: R_1, R_2, R_4, R_5 = F; R_3 = H; X = Br^-
9: R_2, R_3, R_4 = F; R_1, R_5 = H; X = Br^-

The dimethylbenzyl-fluorobenzyl ammonium salts were obtained by the following general approach. In a typical procedure 200 mg dimethylbenzyl amine (1.0 eq., 1.65 mmol) were placed in a round bottom flask and the corresponding amount of the fluorobenzylhalide (1.0 eq.) were directly added. The resulting colorless solid were recrystallized from ethanol.

Dimethylbenzyl-pentafluorobenzylammonium bromide (7)
198 mg (M = 395.03 g/mol, 0.51 mmol, 65%); **mp:** 174 °C
^{1}H-NMR (400 MHz, MeOD): δ (ppm) = 7.62-7.51 (m, 5H, H_{aryl}), 4.84 (s, 2H, H_{benzyl}), 4.71 (s, 2H, H_{benzyl}), 3.03 (s, 6H, CH_3).
^{19}F-NMR (400 MHz, MeOD): δ (ppm) = -138.04 (m, 2F, F_{ortho}), -151.26 (m, 1F, F_{para}), -162.79 (m, 2F, F_{meta}).
MS (ESI): m/z (%) = positive: 316.3 (62, $[M]^+$, $C_{15}H_{13}F_5N^+$), 713.1 (100, $[M_2Br]^+$, $C_{30}H_{26}BrF_{10}N_2^+$).
IR (KBr): ν (cm^{-1}) = 3439 (w), 3381 (w), 3013 (w), 2972 (w), 2324 (w), 2189 (w), 2094 (w), 2029 (w), 1997 (w), 1736 (w), 1660 (m), 1622 (w), 1587 (w), 1507 (vs), 1420 (m), 1363 (w), 1334 (w), 1307 (m), 1217 (w), 1132 (s), 1080 (w), 1044 (m), 979 (s), 936 (s), 915 (m), 860 (s), 820 (m), 780 (m), 759 (s), 720 (s), 679 (w).
Elemental Analysis (%): calculated $C_{16}H_{13}BrF_5N$: C 48.50, H 3.82, N 3.54; found: C 47.82, H 4.03, N 3.49.
Colorless crystals from DMF/Et_2O (see Table S9).

Dimethylbenzyl-2,3,5,6-tetrafluorobenzylammonium bromide (8)
267 mg colorless solid (M = 378.20 g/mol, 0.71 mmol, 85%) **mp:** 190 °C
^{1}H-NMR (300 MHz, MeOD): δ (ppm) = 7.85-7.73 (m, 1H, H_{aryl}), 7.66-7.51 (m, 5H, H_{aryl}), 4.84 (s, 2H, H_{benzyl}), 4.75 (s, 2H, H_{benzyl}), 3.06 (s, 6H, CH_3).
^{19}F-NMR (300 MHz, MeOD): δ (ppm) = -139.03 (m, 4F, F_{ortho}, F_{meta}).
MS (ESI): m/z (%) = positive: 298.2 (100, $[M]^+$, $C_{16}H_{16}F_4N^+$).
IR (KBr): ν (cm^{-1}) = 3061 (w), 3021 (w), 2965 (w), 2323 (w), 2167 (w), 2068 (w), 1987 (w), 1928 (w), 1757 (w), 1611 (w), 1503 (vs), 1455 (m), 1393 (m), 1363 (m), 1334 (w), 1264 (s), 1218 (w), 1178 (s), 1121 (w), 1082 (w), 1037 (m), 985 (s), 930 (m), 858 (vs), 823 (s), 781 (m), 762 (s), 717 (vs), 660 (m).
Elemental Analysis (%): calculated $C_{15}H_{16}BrF_4N$: C 50.81, H 4.26, N 3.70; found: C 50.25, H 4.24, N 3.69.
Colorless crystals from MeOH/Et_2O (see Table S10).

Dimethylbenzyl-3,4,5-trifluorobenzylammonium bromide (9)
177 mg colorless solid (M = 360.21 g/mol, 0.49 mmol, 63%); **mp:** 212 °C
^{1}H-NMR (300 MHz, MeOD): δ (ppm) = 7.63-7.49 (m, 7H, H_{aryl}), 4.67 (s, 2H, H_{benzyl}), 4.67 (s, 2H, H_{benzyl}), 3.01 (s, 6H, CH_3).
^{19}F-NMR (300 MHz, MeOD): δ (ppm) = -135.09 (m, 2F, F_{meta}), -160.06 (m, 1F, F_{para}).
MS (EI, 70eV): m/z (%) = 280.3 (8, $[M]^+$, $C_{16}H_{17}F_3N^+$), 639.5 (100, $[M_2Br]^+$, $C_{32}H_{34}BrF_6N_2^+$).

IR (KBr): ν (cm^{-1}) = 3035 (w), 2969 (m), 2885 (w), 2398 (w), 2318 (w), 2187 (w), 2111 (w), 1904 (w), 1597 (vs), 1468 (vs), 1402 (m), 1326 (s), 1219 (m), 1125 (vs), 1035 (w), 991 (s), 951 (w), 919 (w), 859 (vs), 823 (s), 774 (m), 744 (s), 700 (vs), 659 (w).
Elemental Analysis (%): calculated $C_{16}H_{17}BrF_3N$: C 53.35, H 4.76, N 3.89; found: C 53.23, H 4.61, N 3.85.
Colorless crystals from MeOH/Et_2O (see Table S11).

3,4,5-Trifluorobenzyl-1,4-diazoniabicyclo[2.2.2]-octane chloride (10)

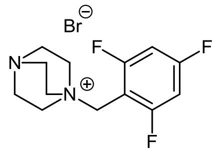

To a solution of 125 mg of 1,4-diazabicyclo[2.2.2]octane (1.0 eq., 1.10 mmol) in 20 mL of chloroform a solution of 250 mg 3,4,5-trifluorobenzyl bromide (1.0eq., 1.10 mmol) in 10 mL chloroform were added. The reactions mixture was stirred for 12 h at room temperature and the resulting precipitate was isolated by filtration, washed with chloroform and dried in vacuo.

199 mg colorless solid (M = 292.10 g/mol, 1.03 mmol, 94%); **mp:** 307 °C
^{1}H-NMR (300 MHz, MeOD): δ (ppm) = 7.44 (t, J = 7.0 Hz, 2H, H_{aryl}), 4.51 (s, 2H, H_{benzyl}), 3.42 (t, J = 6.9 Hz, 6H, CH_2), 4.18 (t, J = 6.9 Hz, 1H, H_{aryl}).
^{19}F-NMR (300 MHz, MeOD): δ (ppm) = -135.00 (m, 2F, F_{meta}), -160.11 (m, 1F, F_{para}).
MS (ESI): m/z (%) = positive: 257.6 (15, $[M]^+$, $C_{13}H_{16}F_3N_2^+$), 549.2 (100, $[M_2Cl]^+$, $C_{26}H_{32}F_6N_4Cl^+$).
IR (KBr): ν (cm^{-1}) = 3382 (m), 3011 (m), 2987 (m), 2918 (m), 2636 (w), 2322 (w), 2112 (w), 1943 (w), 1618 (m), 1533 (vs), 1490 (w), 1453 (s), 1414 (m), 1357 (s), 1324 (m), 1217 (m), 1189 (w), 1149 (m), 1078 (m), 1050 (vs), 1020 (vs), 987 (m), 944 (w), 892 (s), 841 (m), 788 (vs), 743 (s), 667 (m).
Elemental Analysis (%): calculated $C_{13}H_{16}F_6N_2Cl\cdot1/2H_2O$: C 51.75, H 5.68, N 9.28; found: C 51.70, H 5.32, N 9.31.
Colorless crystals from MeOH/EE (see *Table S12*).

2-Amino-pentafluorobenzyl-pyridinium bromide (11)

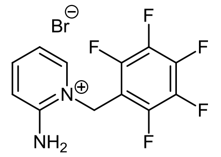

To a solution of 200 mg (1.0 eq., 0.79 mmol, 116 µL) pentafluorobenzyl bromide in 40 mL diethyl ether 74 mg (1.0 eq., 0.79 mmol) 2-amino pyridine were added. The reaction mixture was stirred for 48 h at room temperature. Then the solvent was removed under reduced pressure, washed with aceton and dried in vacuo.

54 mg colorless solid (M = 355.10 g/mol, 0.15 mmol, 19%); **mp:** 237 °C
^{1}H-NMR (300 MHz, MeOD): δ (ppm) = 9.34 (s, 2H, H_{amin}), 7.91 (d, J = 8.6 Hz, 1H, H_{aryl}), 7.65 (m, 1H, H_{aryl}), 7.31 (d, J = 6.8 Hz, 1H, H_{aryl}), 6.75 (t, J = 6.9 Hz, 1H, H_{aryl}), 5.88 (s, 2H, H_{benzyl}).
^{19}F-NMR (300 MHz, MeOD): δ (ppm) = -143.64 (m, 2F, F_{ortho}), -154.28 (m, 1F, F_{para}), -163.53 (m, 2F, F_{meta}).
MS (EI, 70 eV): m/z (%) = 274.2 (51, $[M-H]^+$, $C_{12}H_7F_5N_2^+$), 181.2 (100, $C_7H_2F_5^+$).

IR (KBr): ν (cm^{-1}) = 3193 (m), 3025 (s), 2737 (w), 2690 (w), 2433 (w), 2262 (w), 2089 (w), 1986 (w), 1861 (w), 1739 (m), 1658 (vs), 1582 (vs), 1522 (vs), 1499 (vs), 1453 (m), 1409 (w), 1374 (m), 1337 (w), 1276 (w), 1225 (m), 1127 (s), 1030 (s), 1009 (s), 965 (s), 918 (s), 859 (m), 802 (m), 755 (s), 728 (w), 698 (s), 671 (s).
Elemental Analysis (%): calculated $C_{12}H_8F_5N_2Br$: C 40.59, H 2.27, N 7.89; found: C 40.73, H 2.37, N 7.99.
Colorless crystals from DMF/Et_2O (see Table S13).

3-Amino-pentafluorobenzyl-pyridinium bromide (12)

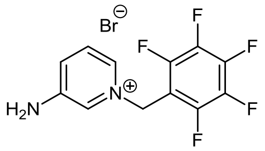

To a solution of 200 mg pentafluorobenzyl bromide (1.0 eq., 0.79 mmol, 116 µL) in 10 mL of tetrahydrofuran a solution of 77 mg 3-aminopyridine (1.0 eq., 0.79 mmol) in 10 mL of tetrahydrofuran was added. The mixture was stirred for 48 h at room temperature. The obtained precipitate was isolated by filtration, washed with tetrahydrofuran and dried in vacuo.
227 mg colorless solid (M = 355.10 g/mol, 0.64 mmol, 78 %); **mp:** 208 °C
^{1}H-NMR (300 MHz, MeOD): δ (ppm) = 8.10 (s, 2H, H_{aryl}),7.67 (m, 2H, H_{aryl}), 5.85 (s, 2H, H_{benzyl}).
^{19}F-NMR (300 MHz, MeOD): δ (ppm) = -142.98 (m, 2F, F_{ortho}), -153.25 (m, 1F, F_{para}), -163.23 (m, 2F, F_{meta}).
MS (EI, 70 eV): m/z (%) = 275.0 (2, [M]$^+$, $C_{12}H_7F_5N_2^+$), 181.0 (100, $C_7H_2F_5^+$).
IR (KBr): ν (cm^{-1}) = 3335 (w), 3288 (w), 3168 (w), 3050 (w), 2946 (w), 2865 (w), 2193 (w), 2094 (w), 1993 (w), 1964 (w), 1660 (w), 1631 (m), 1586 (m), 1503 (vs), 1435 (m), 1377 (w), 1333 (m), 1308 (w), 1284 (w), 1231 (w), 1196 (w), 1160 (m), 1133 (m), 1035 (s), 969 (m), 921 (s), 866 (m), 819 (w), 802 (m), 780 (m), 727 (m), 688 (s).
Elemental Analysis (%): calculated $C_{12}H_8F_5N_2Br$: C 49.59, H 2.27, N 7.89; found: C 49.55, H 2.25, N 7.93.
Colorless crystals from DMF/Et_2O (see Table S14).

Pentafluorobenzyl-4,4'-dipyridylium bromide (13)

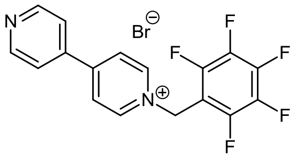

1.000 g Pentafluorobenzyl bromide (3.83 mmol, 1 eq., 580 µL) were added to a solution of 680 mg (4.35 mmol, 1.14 eq.) 4,4'-bipyridine in 100 mL toluene and heated to reflux for 2 h. Then 465 mg 4.4'-bipyridine (2.98 mmol, 0.78 eq.) were added and heated for additional 3.5 h. The colorless precipitate was isolated by filtration, washed with diethyl ether and dried in vacuo.
819 mg colorless solid (M = 417.17 g/mol, 1.96 mmol, 51 %); **mp:** 225.0 °C
^{1}H-NMR-Spektrum ($CDCl_3$, 300 MHz): δ(ppm): 9.59 (d, ^3J = 6.5 Hz, 2H, H_{aryl}); 8.89 (dd, ^3J = 6.2 / 1.7 / 1.6 Hz, 2H, H_{aryl}); 8.41 (d,^3J = 6.5 Hz, 2H, H_{aryl}); 7.70 (dd, ^3J = 6.2 / 1.7 / 1.6 Hz, H_{aryl}); 6.70 (s, 2H, H_{benzyl}).
^{19}F-NMR-Spektrum ($CDCl_3$, 300 MHz): δ(ppm): -138.8 (m, 2F, F_{otho}); -147.8 (m, 1F, F_{para}); -158.4 (m, 2F, F_{meta}).
MS (ESI): m/z (%): 337.1 (1, [M]$^+$, $C_{17}H_{10}F_5N_2^+$); 181.1 (100, $C_7H_2F_5^+$.).
IR (KBr) ṽ(cm-1): 3488 (w), 3385 (w), 3122 (w), 3040 (w), 2990 (w), 2958 (w), 2325 (w),

2192 (w), 2077 (w), 2012 (w), 1972 (w), 1950 (w), 1640 (m), 1598 (m), 1548 (m), 1510 (vs), 1462 (m), 1430 (w), 1407 (m), 1377 (w), 1359 (w), 1340 (w), 1305 (m), 1280 (w), 1220 (m), 1176 (m), 1128 (s), 1032 (s), 964 (m), 923 (s), 870 (w), 836 (w), 814 (s), 767 (m), 746 (m), 714 (m), 669 (m).
Elemental Analysis: calculated $C_{17}H_{10}BrF_5N_2{\cdot}H_2O$: C: 46.92 %H: 2.78 %N: 6.44 %; found: C: 46.94 %H: 2.77 %N: 6.51.
Colorless crystals from MeOH were obtained (see Table S15).

***N*-Methyl-*N*-pentafluorobenzyl-imidazolium bromide (14)**

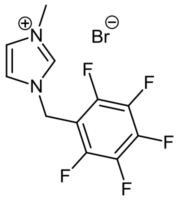

320 mg of *N*-methylimidazole (1.0 eq., 3.90 mmol) were dissolved in 20 mL of chloroform. Then 1.000 g of pentafluorobenzyl bromide (1.0 eq., 3.90 mmol) dissolved in 10 mL of chloroform were added and the reactions mixture was stirred for 12 h at room temperature. The solvent was removed under reduced pressure and the remaining solid was dried in vacuo.
1.32 g colorless solid (M = 343.09 g/mol, 3.85 mmol, 99%); **mp:** 138 °C
^{1}H-NMR (300 MHz, MeOD): δ (ppm) = 7.65 (d, J = 16.1 Hz, 2H, CH), 5.64 (s, 2H, H_{benzyl}), 3.77 (s, 3H, CH_3).
^{19}F-NMR (300 MHz, MeOD): δ (ppm) = -143.91 (m, 2F, F_{otho}), -154.37 (m, 1F, F_{para}), -163.55 (m, 2F, F_{meta}).
MS (ESI): m/z (%) = positive: 263.1 (100, $[M]^+$, $C_{11}H_8F_5N_2^+$).
IR (KBr): ν (cm^{-1}) = 3436 (w), 3126 (w), 3080 (m), 3050 (m), 2974 (m), 2842 (w), 2462 (w), 2237 (w), 2089 (w), 1993 (w), 1770 (w), 1663 (m), 1577 (w), 1548 (m), 1510 (vs), 1448 (m), 1434 (m), 1406 (m), 1349 (w), 1298 (m), 1224 (w), 1164 (vs), 1124 (s), 1025 (s), 969 (s), 921 (s), 888 (m), 790 (s), 727 (w), 707 (m), 678 (m).
Elemental Analysis (%): calculated $C_{11}H_8F_5N_2Br$: C 38.51, H 2.35, N 8.71; found: C 38.59, H 2.40, N 8.39.
Colorless crystals from MeOH/EE (see Table S16).

***N,N-Bis*-pentafluorobenzyl-benzimidazolium bromide (15)**

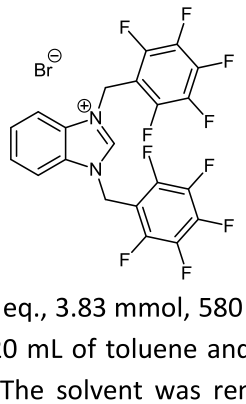

1.000 g Pentafluorobenzyl bromide (2.0 eq., 3.83 mmol, 580 µL) were added to a solution of 225 mg benzimidazole (1.0 eq., 1.90 mmol) in 20 mL of toluene and heated to 110°C for 5 h. The colorless precipitate was removed by filtration. The solvent was removed from the filtrate under reduced pressure and the obtained colorless solid was recrystallized from 50 mL toluene and finally dried in vacuo.
244 mg colorless solid (M = 559.20 g/mol, 0.44 mmol, 23%); **mp:** 194 °C
^{1}H-NMR (300 MHz, MeOD): δ (ppm) = 7.95 (m, 2H, H_{aryl}), 7.79 (m, 2H, H_{aryl}), 5.97 (s, 4H, H_{benzyl}).

^{19}F-NMR (300 MHz, MeOD): δ (ppm) = -143.2 (m, 4F, F_{otho}), -153.9 (m, 2F, F_{para}), -163.4 (m, 4F, F_{meta}).
MS (ESI): m/z (%) = positive: 479.2 (15, $[M]^+$, $C_{21}H_9F_{10}N_2^+$).
IR (KBr): ν (cm^{-1}) = 3109 (w), 3044 (w), 2934 (w), 2866 (m), 2752 (w), 2445 (w), 2085 (w), 2011 (w), 1870 (w), 1737 (w), 1655 (m), 1613 (w), 1556 (m), 1498 (vs), 1451 (w), 1423 (m), 1377 (m), 1307 (m), 1206 (m), 1165 (w), 1129 (s), 1035 (s), 963 (m), 927 (s), 791 (m), 765 (s), 731 (m), 683 (m).
Elemental Analysis (%): calculated $C_{21}H_9F_{10}N_2Br \cdot CH_3C_6H_5$: C 47.51, H 1.99, N 4.75; found: C 47.10, H 2.22, N 4.82.
Colorless crystals from MeOH/EE (see Table S17).

1,4-Dimethyl-4-pentafluorobenzyl piperazine (16)

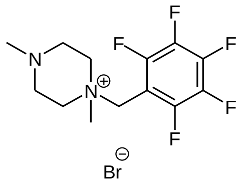

88 mg Dimethyl piperazine (1.0 eq., 0.77 mmol, 105 µL) were added to a solution of 200 mg pentafluorobenzyl bromide (1.0 eq, 0.77 mmol, 116 µL) in 10 mL of toluene and stirred for 12 h at room temperature. The colorless precipitate was isolated by filtration and dried in vacuo.
130 mg colorless solid (M = 370.04 g/mol, 0.35 mmol, 51%); **mp:** 270 °C under combustion
^{1}H-NMR (300 MHz, MeOD): δ (ppm) = 4.82 (s, 2H, H_{benzyl}), 3.62 (m, 4H, CH_2), 3.18 (s, 2H, CH_3), 2.92 (m, 2H, CH_2), 2.76 (m, 2H, CH_2), 2.41 (s, 3H, CH_3).
^{19}F-NMR (300 MHz, MeOD): δ (ppm) = -137.27 (m, 2F, F_{ortho}), -151.03 (m, 1F, F_{para}), -160.70 (m, 2F, F_{meta}).
MS (ESI): m/z (%) = positive: 295.5 (100, $[M]^+$, $C_{13}H_{16}F_5N_2^+$).
IR (KBr): ν (cm^{-1}) = 2969 (w), 2942 (w), 2804 (m), 2168 (w), 1983 (w), 1740 (w), 1656 (m), 1506 (vs), 1470 (s), 1442 (m), 1393 (m), 1333 (w), 1309 (m), 1288 (m), 1236 (w), 1209 (w), 1136 (s), 1085 (m), 1058 (m), 1002 (vs), 944 (vs), 908 (s), 825 (w), 791 (m), 751 (w), 689 (w).
Elemental Analysis (%): calculated $C_{13}H_{16}BrF_5N_2$: C 41.74, H 4.31, N 7.49; found: C 41.80, H 4.23, N 7.42.
Two sets of colorless crystals from MeOH/Et_2O (see Table S18 and Table S19).

2-Hydroxy-*N,N*-dimethyl-*N*-pentafluorobenzyl-ethaneammonium bromide (17)

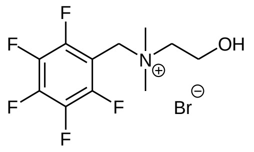

200 mg pentafluorobenzyl bromide (1.0 eq., 0.77 mmol, 116 µL) were added to a solution of 69 mg (1.0 eq., 0.77 mmol, 77 µL) *N,N,*-dimethylamino ethanol in 20 mL of diethyl ether and stirred for 24 h at room temperature. The solvent was removed under reduced pressure and the remaining colorless solid was dried in vacuo.
164 mg colorless solid (M = 349.1 g/mol, 0.47 mmol, 61 %); **mp:** 194 °C
^{1}H-NMR (300 MHz, MeOD): δ (ppm) = 4.74 (s, 2H, H_{benzyl}), 4.00 (t, J = 4.7 Hz, 2H, CH_2), 3.60 (t, J = 4.7 Hz, 2H, CH_2), 3.15 (s, 6H, CH_3).
^{19}F-NMR (300 MHz, MeOD): δ (ppm) = -137.92 (m, 2F, F_{ortho}), -151.34 (m, 1F, F_{para}), -162.78 (m, 2F, F_{meta}).
MS (ESI): m/z (%) = positive: 270.6 (100, $[M]^+$, $C_{11}H_{13}F_5NO^+$).

IR (KBr): ν (cm^{-1}) = 3295 (m), 3009 (w), 2962 (w), 2846 (w), 2655 (w), 2185 (w), 2089 (w), 1978 (w), 1663 (m), 1528 (s), 1508 (vs), 1485 (s), 1451 (m), 1432 (m), 1381 (m), 1350 (w), 1332 (w), 1308 (m), 1261 (m), 1220 (w), 1164 (w), 1136 (s), 1088 (m), 1035 (s), 995 (m), 976 (s), 952 (vs), 899 (s), 844 (m), 800 (m), 765 (w), 726 (w), 677 (m).
Elemental Analysis (%): calculated $C_{11}H_{13}BrF_5NO$: C 37.73, H 3.74, N 4.00; found: C 37.60, H 3.79, N 3.85.
Colorless crystals from MeOH/Et_2O (see Table S20).

Tetramethyl-*bis*-(pentafluorobenzyl)ethylendiammonium dibromide (18)

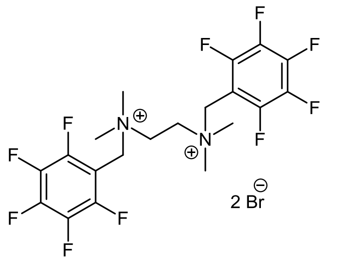

400 mg Pentafluorobenzyl bromide (2.0 eq., 1.54 mmol, 230 µL) were dissolved in 10 mL of acetonitrile. To this solution 89 mg tetramethylethylene diamine (1.0 eq., 0.77 mmol, 115 µL) were added and the mixture was stirred for 48 h at room temperature. The colorless precipitate was isolated by filtration and washed with three portions of ethyl acetate. Finally the product was dried under vacuo.
392 mg colorless solid (M = 635.98 g/mol, 0.62 mmol, 80%); **mp:** 205 °C
^{1}H-NMR (300 MHz, D_2O): δ (ppm) = 4.80 (s, 4H, H_{benzyl}), 4.20 (s, 4H, CH_2), 3.19 (s, 12H, CH_3).
^{19}F-NMR (300 MHz, D_2O): δ (ppm) = -136.45 (m, 2F, F_{ortho}), -146.86 (m, 1F, F_{para}), -159.68 (m, 2F, F_{meta}).
MS (ESI): m/z (%) = positive: 559.0 (100, $[M+Br]^+$, $C_{20}H_{20}BrF_{10}N_2^+$).
IR (KBr): ν (cm^{-1}) = 3007 (w), 2954 (w), 2049 (w), 1982 (w), 1663 (m), 1508 (vs), 1476 (m), 1443 (m), 1403 (w), 1381 (w), 1357 (w), 1331 (w), 1308 (m), 1234 (w), 1135 (s), 1035 (s), 969 (vs), 924 (w), 904 (m), 892 (m), 876 (m), 816 (m), 766 (w), 721 (m), 682 (m).
Elemental Analysis (%): calculated $C_{20}H_{20}Br_2F_{10}N_2$: C 37.64, H 3.16, N 4.39; found: C 37.60, H 3.25, N 4.38.
Three sets of colorless crystals from MeOH/Et_2O (see Table S21, Table S22 and Table S23).
***N*-Pentafluorobenzyl-thiomorpholine (19)**

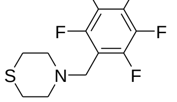

To a solution of 200 mg thiomorpholine (1.0 eq., 1.94 mmol) in 10 mL of chloroform a solution of 500 mg pentafluorobenzyl bromide (1.0 eq., 1.94 mmol) in 10 mL of chloroform was added. The mixture was stirred for 12 h at room temperature in the presence of K_2CO_3 (800 mg, 3.0 eq., 5.82 mmol). The K_2CO_3 was removed by filtration and the solvent was removed under reduced pressure. The remaining residue was dried in vacuo to a colorless solid.
320 mg colorless solid (M = 362.97 g/mol, 1.13 mmol, 58 %); **mp:** 178 °C
^{1}H-NMR ($CDCl_3$, 300 MHz, a9021240): δ (ppm): 3,67(s, 2H, H_{benzyl}); 2,74 (t, ^3J = 5,5 Hz, 4H, CH_2); 2,65 (t, ^3J = 5,5 Hz, 4H, CH_2).
^{19}F-NMR ($CDCl_3$, 300 MHz, a9021240): δ (ppm): -141,0 (m, 2F, F_{ortho}); -154,7 (m, 1F, F_{para}); -162,0 (m, 2F, F_{meta}).
MS (ESI): m/z (%): 284,1 (100, M + H^+, $C_{11}H_{11}F_5NS^+$).

IR (KBr) $\tilde{\nu}(cm^{-1})$: 2944 (m), 2879 (w), 2819 (m), 2753 (w), 2702 (w), 2643 (w), 2168 (w), 2079 (w), 2011 (w), 1871 (w), 1707 (w), 1653 (m), 1585 (w), 1517 (s), 1493 (vs), 1456 (m), 1419 (m), 1405 (w), 1384 (w), 1324 (s), 1275 (w), 1209 (w), 1170 (w), 1126 (m), 1107 (s), 1034 (vs), 989 (s), 961 (s), 935 (m), 909 (s), 803 (w), 768 (w), 745 (s), 690 (m), 670 (m).
Elemental Analysis (%): calculated $C_{11}H_{10}F_5NS$: C 46.64, H 3.56, N 4.94; found: C 46.43, H 3.37, N 4.90.
Colorless Crystals of the methylated bromide salt by slow diffusion of HBr into a solution of **19** in chloroform/methanol (see Table S24).

(2-Dimethylaminoethyl)dimethyl(pentafluorobenzyl)ammonium chloride (20)

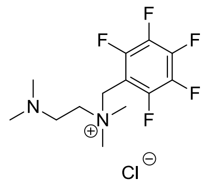

To a solution of *N*,*N*,*N*,*N*-tetramethylethylenediamine (0.492 g, 4.20 mmol) in chloroform (20 mL) was added an equimolar amount of pentafluorobenzyl chloride (900 mg, 4.20 mmol). After stirring the solution for 24 h at room temperature the obtained precipitate was filtered off and dried in vacuo.
1.115 g colorless solid (M = 332.11 g/mol, 3.36 mmol, 80 %); **mp:** 109 °C
^{1}H-NMR (CD_3OD, 300 MHz): δ (ppm): 4,90 (s, 2H, $C_6F_5CH_2$); 3,65 (t, ^3J = 6,1 Hz, 2H, CH_2); 3,19 (s, 6H, CH_3); 2,88 (t, ^3J = 6,1 Hz, CH_2); 2,33 (s, 6H, CH_3)
^{19}F-NMR (CD_3OD, 300 MHz): δ (ppm): -138,0 (d, ^3J = 18 Hz, 2F, F_{ortho}); -151,3 (t, ^3J = 20 Hz, 1F, F_{para}); -162,8 (t, ^3J = 18 Hz, 2F, F_{meta}).
MS (ESI): m/z (%): 297,2 (100, M^+, $C_{13}H_{18}F_5N_2^+$).
IR (KBr) $\tilde{\nu}(cm^{-1})$: 3466 (w), 3008 (w), 2979 (w), 2936 (m), 2840 (w), 2783 (w), 2442 (w), 2168 (w), 2030 (w), 1923 (w), 1728 (w), 1659 (m), 1506 (vs), 1460 (m), 1391 (w), 1368 (m), 1308 (m), 1270 (w), 1234 (w), 1161 (w), 1132 (s), 1082 (w), 1034 (s), 981 (s), 954 (m), 917 (m), 840 (m), 786 (w), 747 (vs), 664 (m).
Elemental Analysis (%): calculated $C_{13}H_{18}ClF_5N_2$: C 46.93, H 5.45, N 8.42; found: C 46.80, H 5.37, N 8.90.
Colorless Crystals wet MeOH/EE (see Table S25).

II. Crystallographic data

General

Single crystal X-ray data were collected at 123(2) K using a Bruker-Nonius KappaCCD diffractometer with APEX-II detector and graphite monochromatized Mo-Kα (λ = 0.71073 Å) radiation. COLLECT[10a] software was used for the data collection (θ and ω scans) and DENZO-SMN[10b] for the processing. The structures were solved by direct methods with SIR2004[10c] and refined by full-matrix least-squares methods with WinGX-software,[10d] which utilizes the SHELXL-97 module.[10e] Lorentzian polarization correction was applied on all data and absorption effects were corrected with multi-scan method (SADABS[10f]). All C–H hydrogen positions were calculated and refined as riding atoms, except for formyl H-atoms in DMF molecules. Hydrogen atoms bonded to O, N (except in **44**) or formyl C were found from Fourier maps and restrained (by DFIX, s = 0.02) to a distance of 0.84 Å from O, 0.88 (amide, aromatic) or 0.91 Å from N or 0.95 Å from formyl C atoms. In structure **44** H atoms bonded to N were refined as riding atoms. Thermal parameters of H-atoms were set to 1.2 times that of the C and N or 1.5 times of O atom parameters. Crystallographic data and parameters are summarized in Tables S2-S26.

Table S1. Representative views of the analyzed anion-π contacts, the corresponding CCDC numbers and the position number for the relative position of the anion in respect to the electron-deficient arene (see Table S27).

#	Structure	CCDC # / Crystallographic data	Pos. #
1	a) 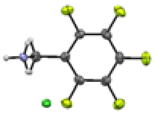 b) 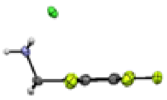	CCDC-866136[1]	Cl12
2	a) 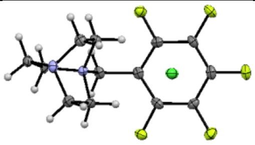 b) 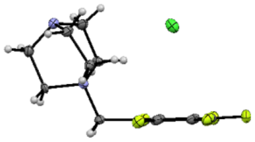	CCDC-752833[2]	Cl17
3	a) 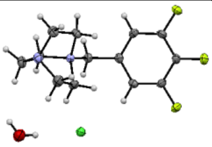 b) 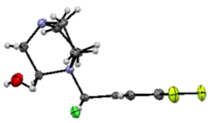	CCDC-1005267 Table S12	Cl16
4	a) 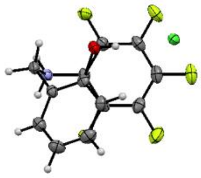 b) 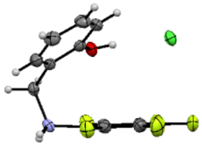	CCDC-883887[3]	Cl13
5	a) 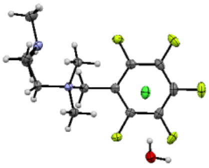 b) 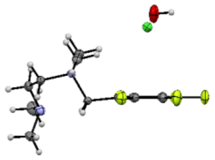	CCDC-1005268 Table S25	Cl14

6	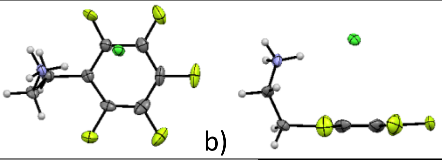 a) b)	CCDC-866137[1]	Cl15
7	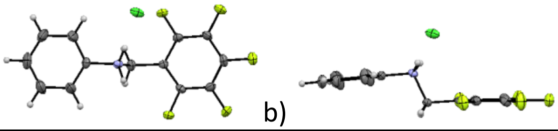 a) b)	CCDC-775333[4]	Cl18
8	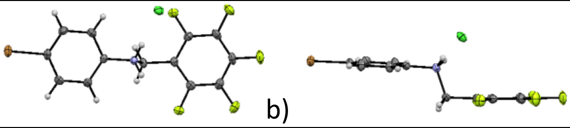 a) b)	CCDC-745350[4]	Cl19
9	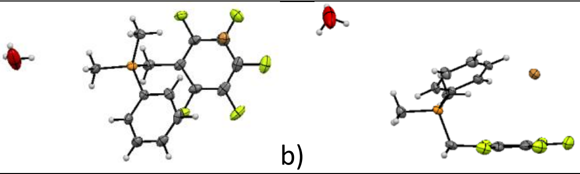 a) b)	CCDC-795076[2]	Br20
	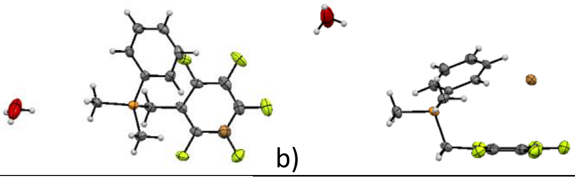 a) b)		Br21
10	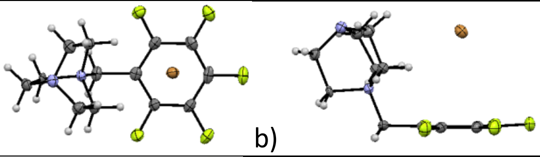 a) b)	CCDC-752834[2]	Br72
11	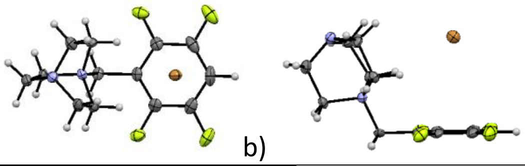 a) b)	CCDC-817432[5]	Br71
12	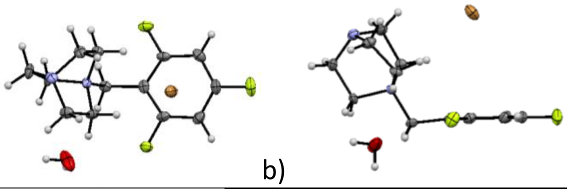 a) b)	CCDC-817433[5]	Br32
	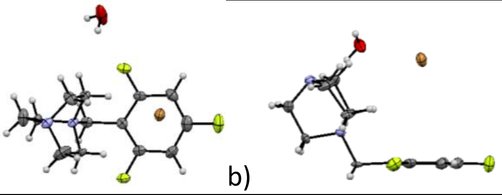 a) b)		
13	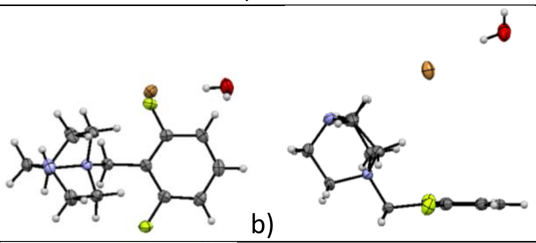 a) b)	CCDC-817434[5]	Br40
14	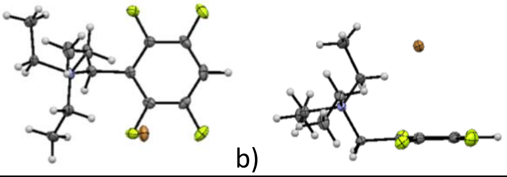 a) b)	CCDC-1005269 Table S2	Br35

15	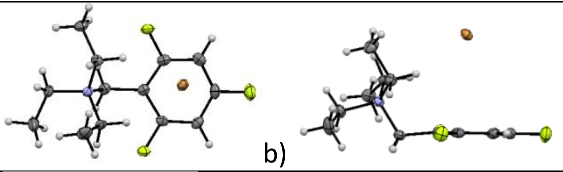 a) b)	CCDC-1005270 Table S5	Br31
16	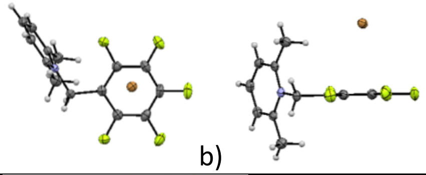 a) b)	CCDC-752839[2]	Br61
17	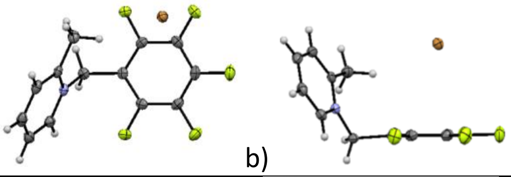 a) b)	CCDC-752838[2]	Br62
18	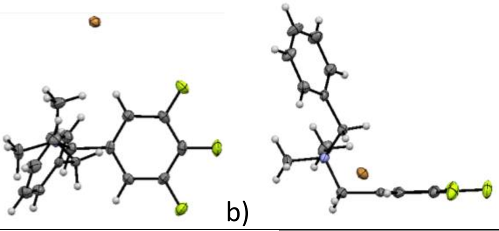 a) b)	CCDC-1005271 Table S11	Br22
19	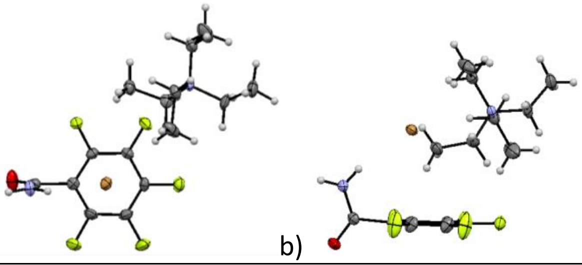 a) b)	CCDC-872224[6]	Br23
20	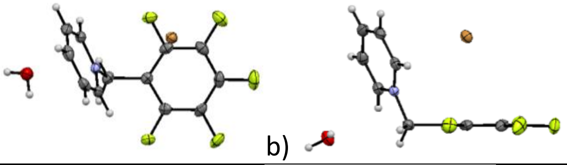 a) b)	CCDC-936265[7]	Br25
21	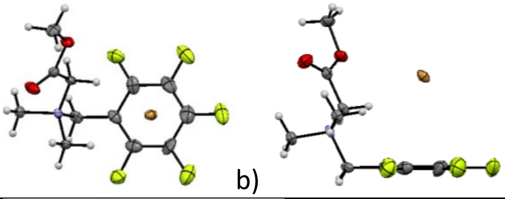 a) b)	CCDC-868496[8]	Br26
22	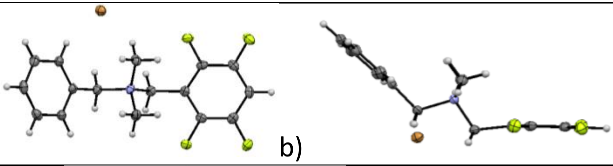 a) b)	CCDC-1005272 Table S10	Br27
23	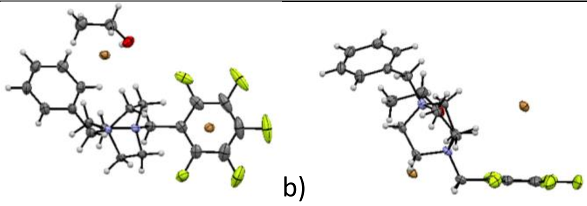 a) b) 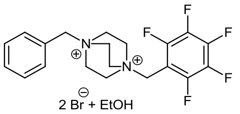	See reference[9]	Br30

24	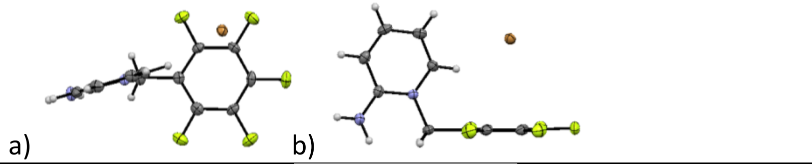	CCDC-1005273<br>Table S13	Br34
25	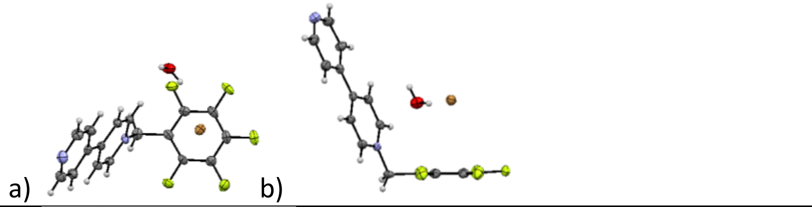	CCDC-1005274<br>Table S15	Br36
26	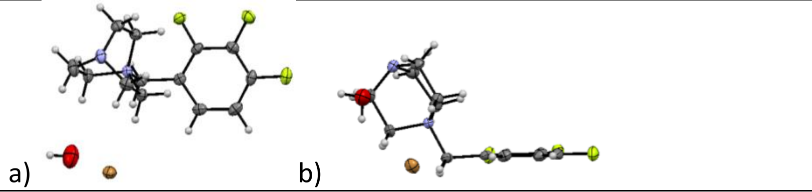	CCDC-817436[5]	Br37
27	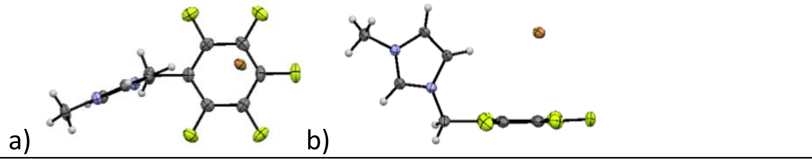	CCDC-1005275<br>Table S16	Br39
28	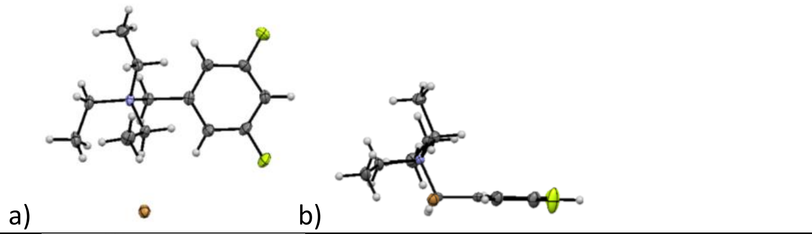	CCDC-1005276<br>Table S7	Br41
29	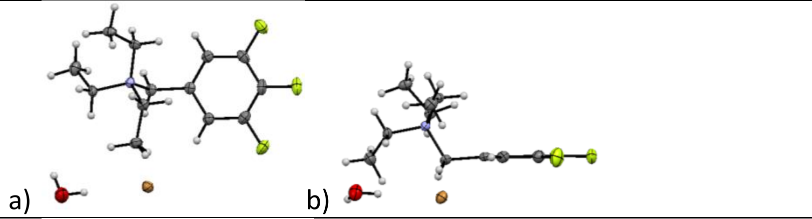	CCDC-1005277<br>Table S6	Br42
30	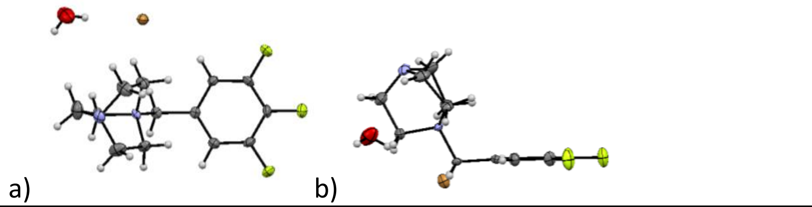	CCDC-817435[5]	Br43
31	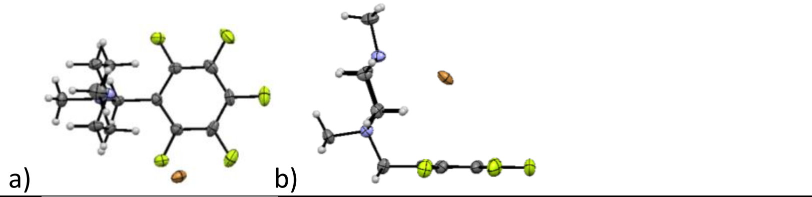	CCDC-1005278<br>Table S18	Br44
32	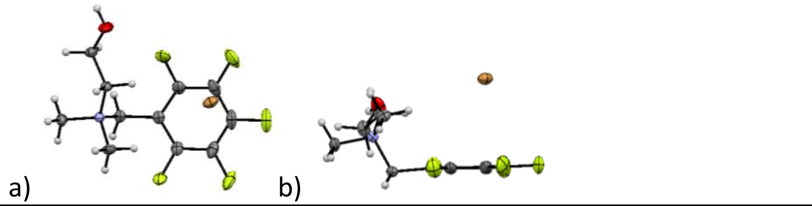	CCDC-1005279<br>Table S20	Br47

33	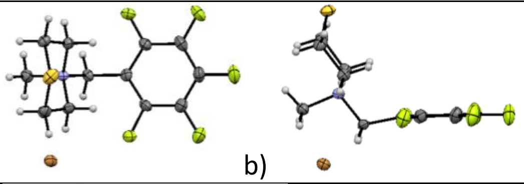 a) b)	CCDC-1005280 Table S24	Br56
34	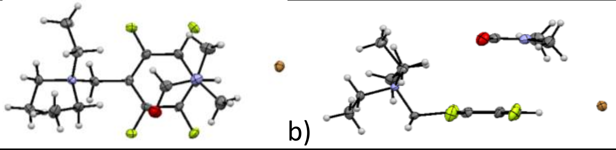 a) b)	CCDC-1005281 Table S3	Br57
35	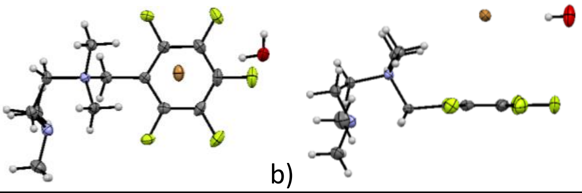 a) b)	CCDC-752832[2]	Br60
36	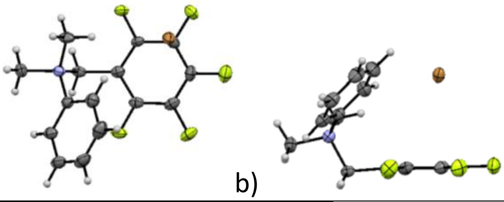 a) b)	CCDC-752831[2]	Br65
37	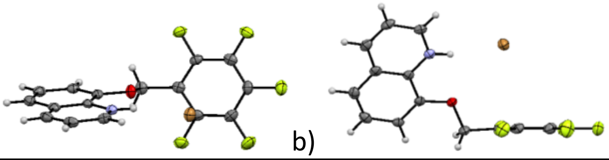 a) b)	CCDC-779160[10]	Br68
38	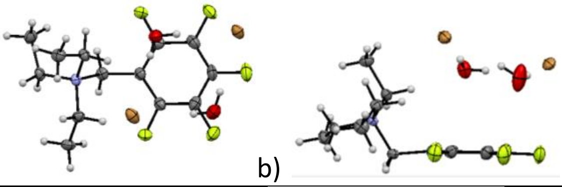 a) b)	CCDC-752830[2]	Br58 Br59
39	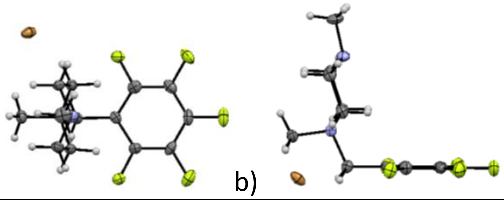 a) b) 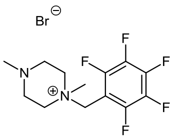	CCDC-1005282 Table S19	Br24
40	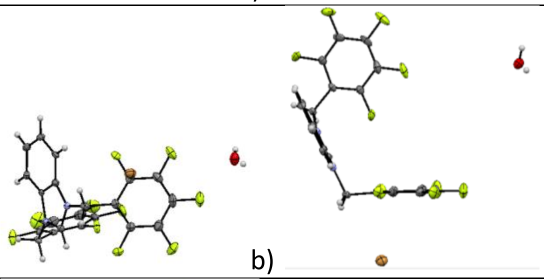 a) b) 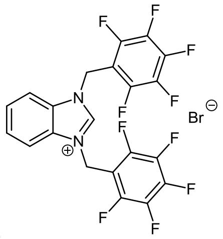	CCDC-1005283 Table S17	Br69
41	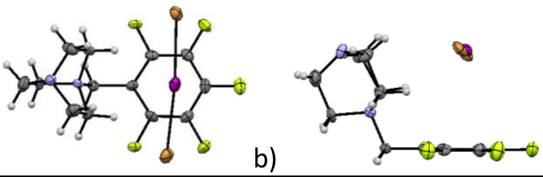 a) b)	CCDC-831306[10]	Br76 Br77 I78

	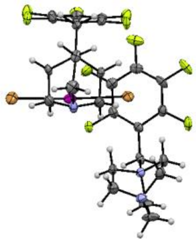 c)		
42	a) 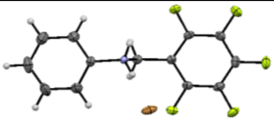 b) 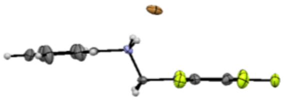	CCDC-745349[4]	Br75
43	a) 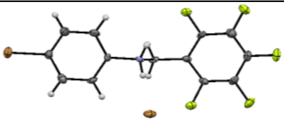 b) 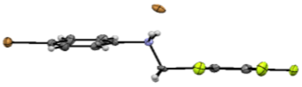	CCDC-745351[4]	Br74
44	a) 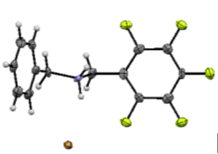 b) 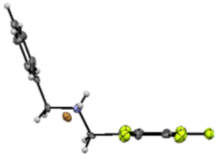	CCDC-1005284 Table S9	Br73
45	a) 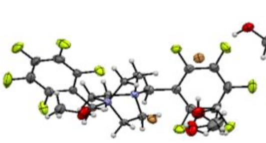 b) 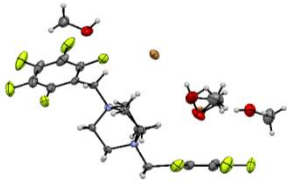 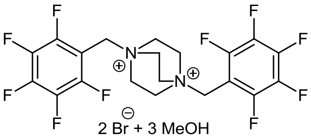	CCDC-752837[2]	Br70
46	a) 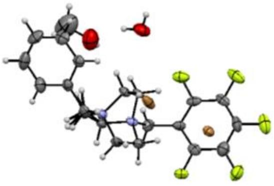 b) 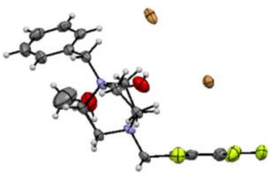 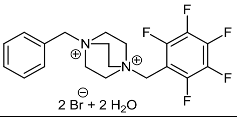	See reference[9]	Br50
47	a) 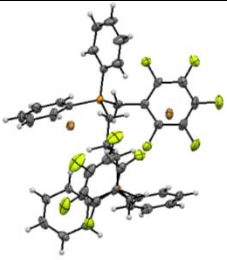 b) 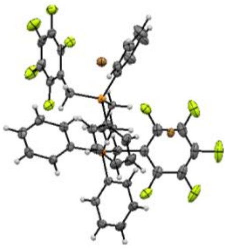 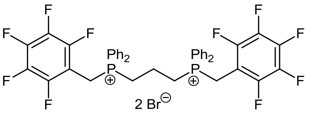	CCDC-774614[10]	Br63 Br64
48	a) 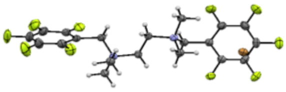 b) 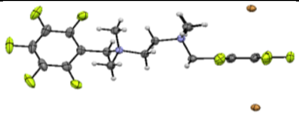	CCDC-967089 Table S21	Br51 Br52

49	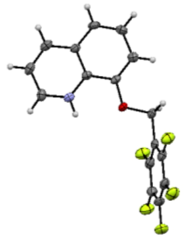 a) 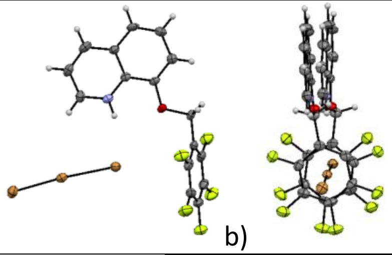 b)	CCDC-779161[10]	Br66<br>Br67
50	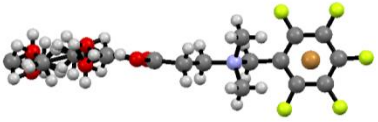 a) 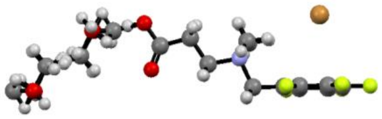 b)	CCDC-868497[8]	Br55
51	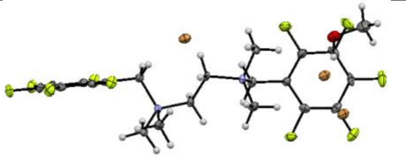 a) 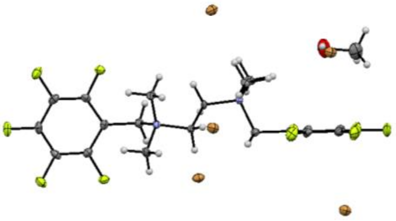 b)	CCDC-1005285<br>Table S22	Br53<br>Br54
52	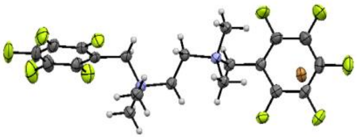 a) 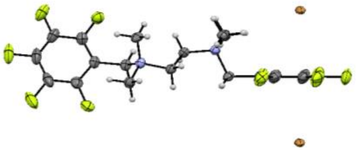 b)	CCDC-967097<br>Table S23	Br38
53	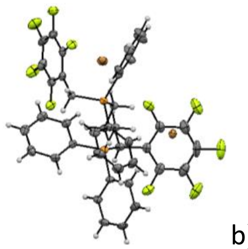 a) 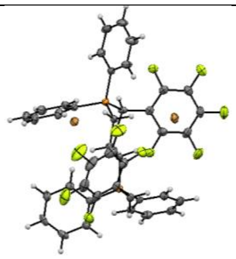 b) 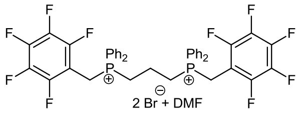	CCDC-915604[11]	Br28<br>Br29
54	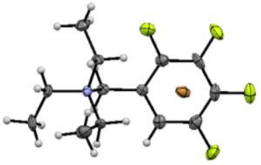 a) 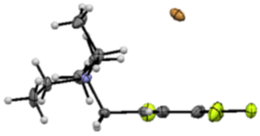 b)	CCDC-1005286<br>Table S4	Br48

	a) 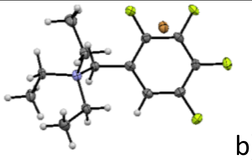 b) 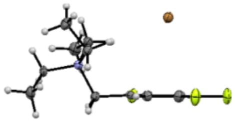		Br49
55	a) 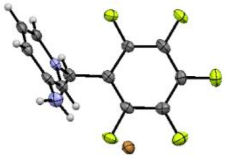 b) 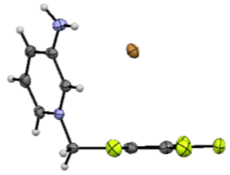	CCDC-1005287 Table S14	Br33
56	a) 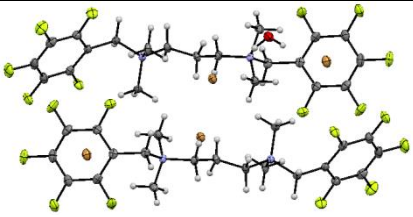 b) 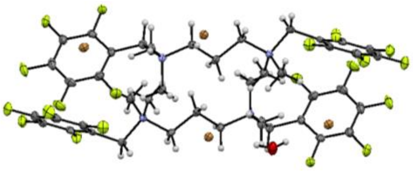	See reference[9]	Br45 Br46
57	a) 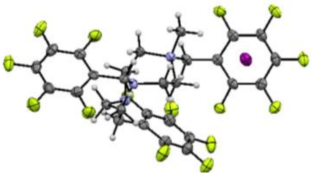 b) 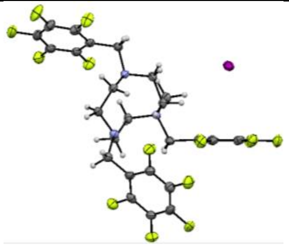 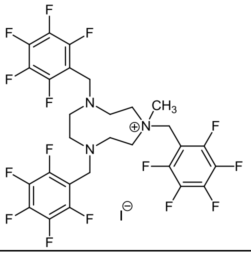	See reference[9]	I79
58	a) 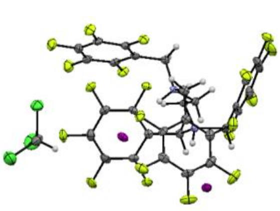 b) 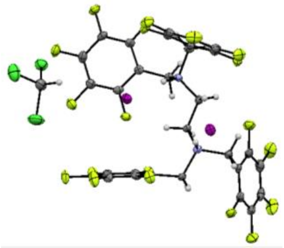 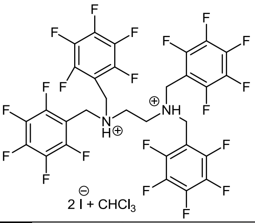	See reference[9]	I85
59	a) 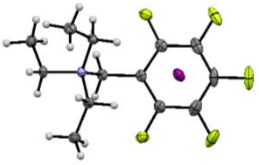 b) 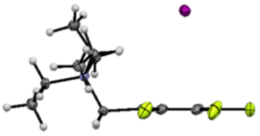	CCDC-1005288 Table S8	I83

60	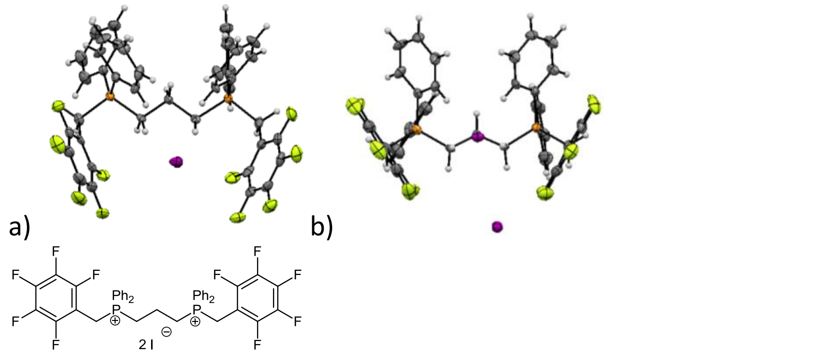	CCDC-774616[10]	I80<br>I81
61	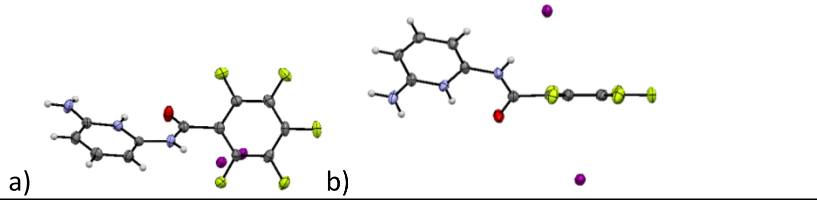	CCDC-1005289<br>Table S26	I86<br>I87
62	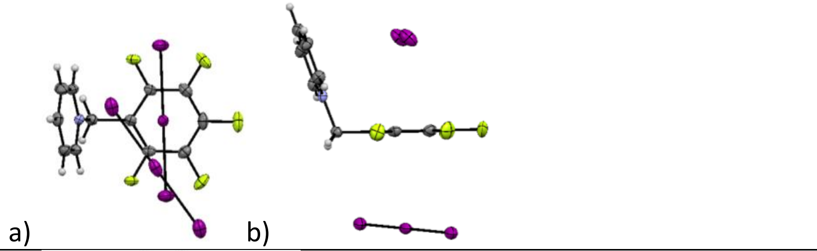	CCDC-936266[7]	I89
63	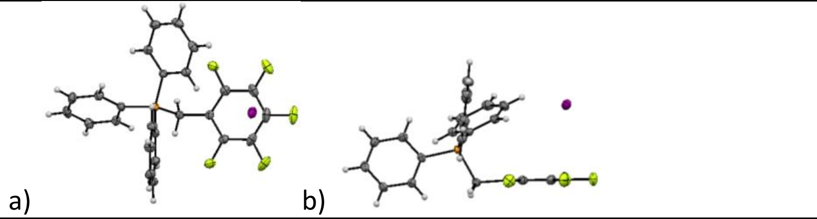	CCDC-915599[11]	I88
64	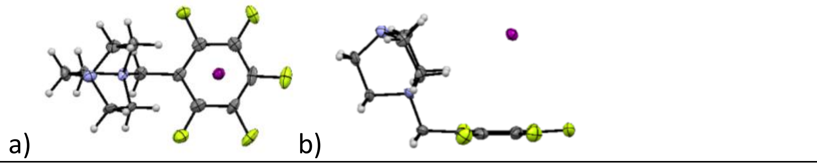	CCDC-752835[2]	I95
65	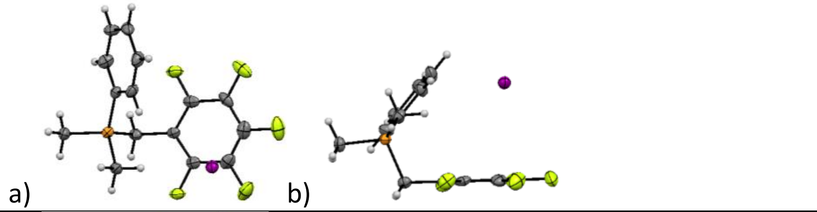	CCDC-795077[12]	I93
	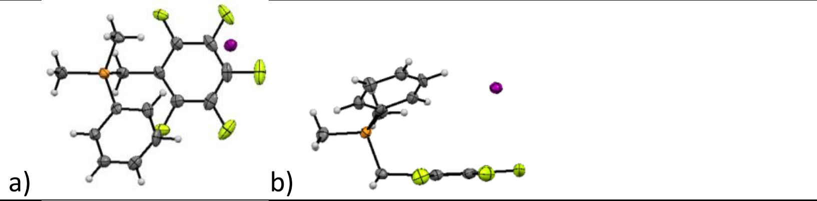		I94
66	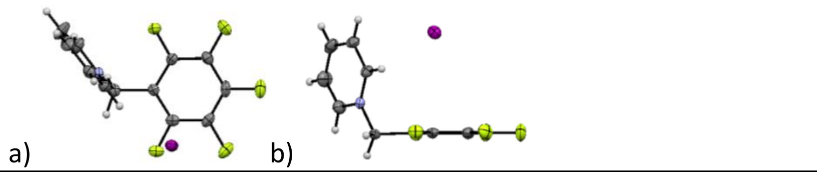	CCDC-936267[7]	I84

	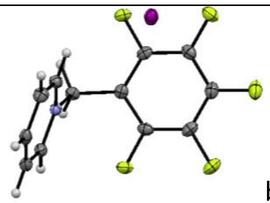 a)<br>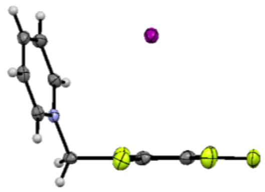 b)		
67	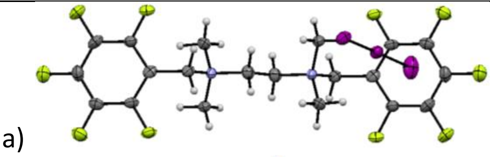 a) b)<br>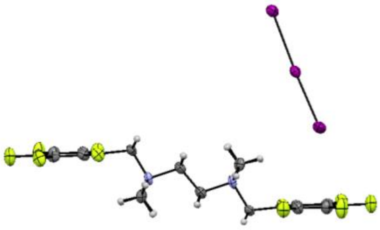	CCDC-967130[13]	I82
68	a)<br>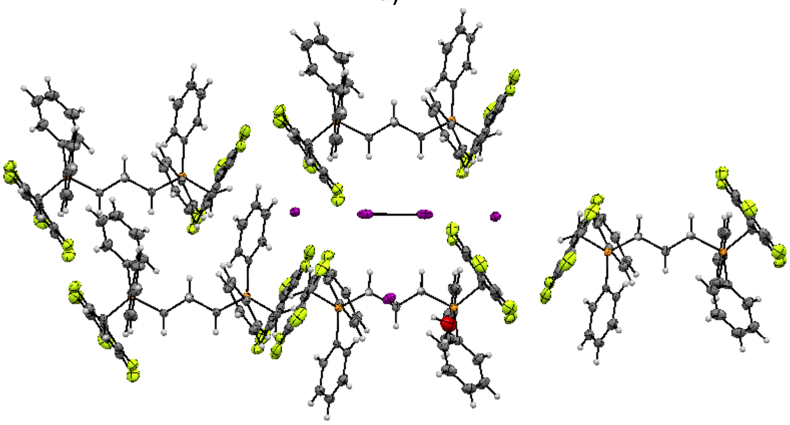<br>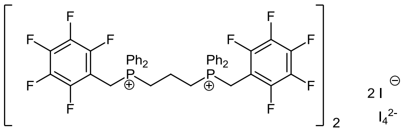	CCDC-774617[10]	I90<br>I91<br>I92
69	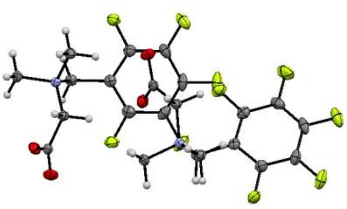 a)<br>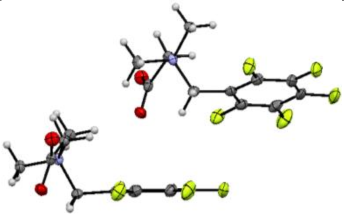 b)	CCDC-868498[8]	Co101
70	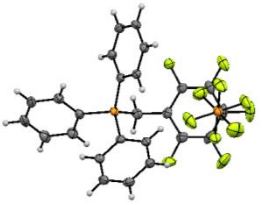 a)<br>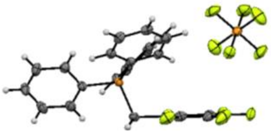 b)	CCDC-915601[11]	Co103
71	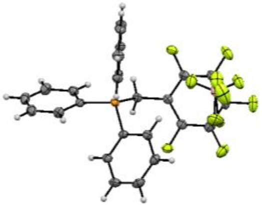 a)<br>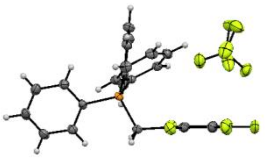 b)	CCDC-915600[11]	Co102

72	a) 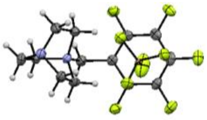 b) 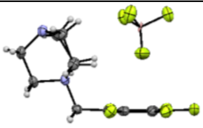	CCDC-831302[14]	Co108
	a) 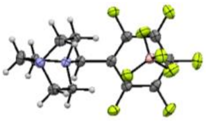 b) 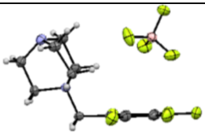		Co109
73	a) 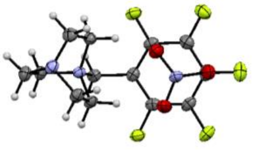 b) 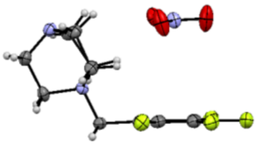	CCDC-831304[14]	Co106
	a) 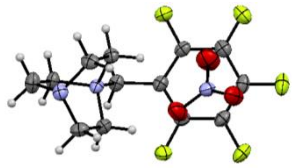 b) 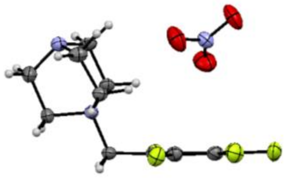		Co107
74	a) 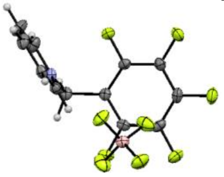 b) 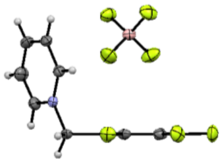	CCDC-936268[7]	Co104
	a) 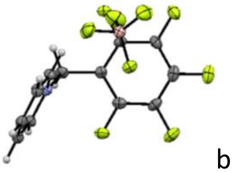 b) 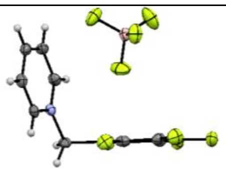		Co105
75	a) 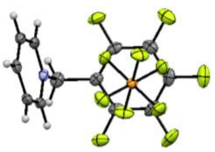 b) 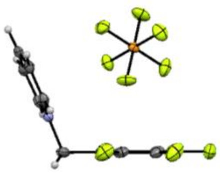	CCDC-936269[7]	Co110
76	a) 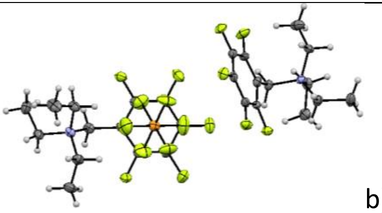 b) 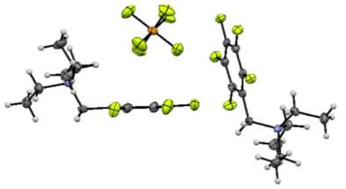	CCDC-831303[14]	Co96
77	a) 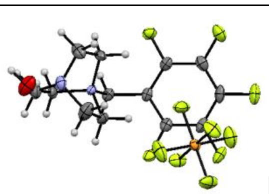 b) 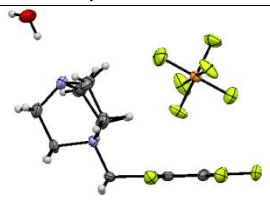	CCDC-752836[14]	Co112
78	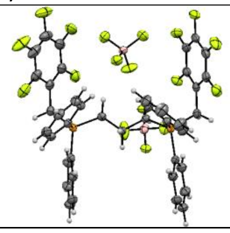 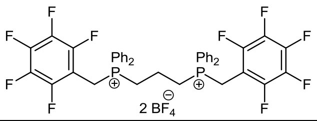	CCDC-915605[11]	Co97<br>Co98

79	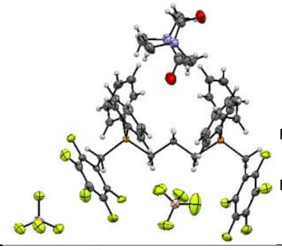 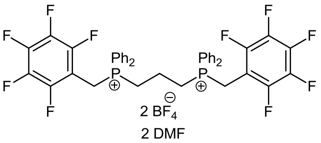	CCDC-915606[11]	Co99<br>Co100
80	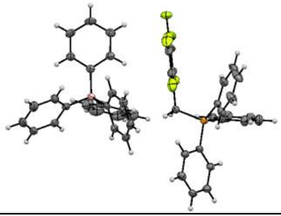 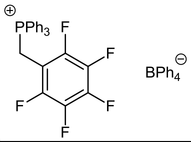	CCDC-795078[12]	Co111

Crystal data of the present paper:

Table S2. Crystal data and structure refinement for (1A).

Empirical formula	$C_{13}H_{18}BrF_4N$	
Formula weight	344.19	
Temperature	123(2) K	
Wavelength	0.71073 Å	
Crystal system	Monoclinic	
Space group	$P2_1/c$	
Unit cell dimensions	a = 12.9686(2) Å	α= 90°.
	b = 8.1122(2) Å	β= 110.7290(10)°.
	c = 14.4871(3) Å	γ = 90°.
Volume	1425.44(5) $Å^3$	
Z	4	
Density (calculated)	1.604 Mg/m^3	
Absorption coefficient	2.915 mm^{-1}	
F(000)	696	
Crystal size	0.30 x 0.22 x 0.20 mm^3	
Theta range for data collection	3.01 to 25.25°.	
Index ranges	-15<=h<=15, -9<=k<=9, -17<=l<=17	
Reflections collected	4641	
Independent reflections	2573 [R(int) = 0.0161]	
Completeness to theta = 25.25°	99.7 %	
Absorption correction	Semi-empirical from equivalents	
Max. and min. transmission	0.5933 and 0.4751	
Refinement method	Full-matrix least-squares on F^2	
Data / restraints / parameters	2573 / 0 / 172	
Goodness-of-fit on F^2	1.022	
Final R indices [I>2σ(I)]	R1 = 0.0242, wR2 = 0.0539	
R indices (all data)	R1 = 0.0322, wR2 = 0.0574	
Largest diff. peak and hole	0.267 and -0.287 e.$Å^{-3}$	

Table S3. Crystal data and structure refinement for (1B).

Empirical formula	$C_{16}H_{25}BrF_4N_2O$	
Formula weight	417.29	
Temperature	123(2) K	
Wavelength	0.71073 Å	
Crystal system	Monoclinic	
Space group	$P2_1$	
Unit cell dimensions	a = 7.6426(2) Å	α= 90°.
	b = 11.6845(3) Å	β= 107.3460(10)°.
	c = 10.7767(3) Å	γ = 90°.
Volume	918.59(4) $Å^3$	
Z	2	
Density (calculated)	1.509 Mg/m^3	
Absorption coefficient	2.281 mm^{-1}	
F(000)	428	
Crystal size	0.30 x 0.20 x 0.08 mm^3	
Theta range for data collection	2.64 to 25.00°.	
Index ranges	-9<=h<=7, -13<=k<=13, -12<=l<=12	
Reflections collected	11604	
Independent reflections	3178 [R(int) = 0.0473]	
Completeness to theta = 25.00°	99.9 %	
Absorption correction	Semi-empirical from equivalents	
Max. and min. transmission	0.8386 and 0.5477	
Refinement method	Full-matrix least-squares on F^2	
Data / restraints / parameters	3178 / 2 / 223	
Goodness-of-fit on F^2	1.071	
Final R indices [I>2σ(I)]	R1 = 0.0295, wR2 = 0.0620	
R indices (all data)	R1 = 0.0347, wR2 = 0.0640	
Absolute structure parameter	0.063(8)	
Largest diff. peak and hole	0.229 and -0.267 e.$Å^{-3}$	

Restraints: floating origin, DFIX (s = 0.02)

Table S4. Crystal data and structure refinement for (2).

Empirical formula	$C_{13}H_{18}BrF_4N$	
Formula weight	344.19	
Temperature	123(2) K	
Wavelength	0.71073 Å	
Crystal system	Orthorhombic	
Space group	$Pca2_1$	
Unit cell dimensions	a = 14.1641(2) Å	α= 90°.
	b = 18.5407(3) Å	β= 90°.
	c = 10.9050(2) Å	γ = 90°.
Volume	2863.79(8) $Å^3$	
Z	8	
Density (calculated)	1.597 Mg/m^3	
Absorption coefficient	2.901 mm^{-1}	
F(000)	1392	
Crystal size	0.20 x 0.18 x 0.04 mm^3	
Theta range for data collection	2.60 to 25.25°.	
Index ranges	-14<=h<=17, -22<=k<=22, -13<=l<=13	
Reflections collected	35549	
Independent reflections	5158 [R(int) = 0.0545]	
Completeness to theta = 25.25°	99.8 %	
Absorption correction	Semi-empirical from equivalents	
Max. and min. transmission	0.8928 and 0.5946	
Refinement method	Full-matrix least-squares on F^2	
Data / restraints / parameters	5158 / 1 / 344	
Goodness-of-fit on F^2	1.021	
Final R indices [I>2σ(I)]	R1 = 0.0285, wR2 = 0.0583	
R indices (all data)	R1 = 0.0346, wR2 = 0.0608	
Absolute structure parameter	0.090(7)	
Largest diff. peak and hole	0.547 and -0.273 e.$Å^{-3}$	

Restraint: floating origin

Table S5. Crystal data and structure refinement for (3).

Empirical formula	$C_{13}H_{19}BrF_3N$	
Formula weight	326.20	
Temperature	123(2) K	
Wavelength	0.71073 Å	
Crystal system	Monoclinic	
Space group	$P2_1/n$	
Unit cell dimensions	a = 7.75180(10) Å	α= 90°.
	b = 10.4214(2) Å	β= 100.5560(10)°.
	c = 17.7318(3) Å	γ = 90°.
Volume	1408.21(4) $Å^3$	
Z	4	
Density (calculated)	1.539 Mg/m^3	
Absorption coefficient	2.936 mm^{-1}	
F(000)	664	
Crystal size	0.30 x 0.20 x 0.16 mm^3	
Theta range for data collection	2.71 to 25.02°.	
Index ranges	-9<=h<=9, -12<=k<=11, -20<=l<=21	
Reflections collected	8157	
Independent reflections	2495 [R(int) = 0.0229]	
Completeness to theta = 25.02°	99.9 %	
Absorption correction	Semi-empirical from equivalents	
Max. and min. transmission	0.6509 and 0.4729	
Refinement method	Full-matrix least-squares on F^2	
Data / restraints / parameters	2495 / 0 / 163	
Goodness-of-fit on F^2	1.036	
Final R indices [I>2σ(I)]	R1 = 0.0216, wR2 = 0.0486	
R indices (all data)	R1 = 0.0267, wR2 = 0.0501	
Largest diff. peak and hole	0.271 and -0.249 e.$Å^{-3}$	

Table S6. Crystal data and structure refinement for (4).

Empirical formula	$C_{13}H_{21}BrF_3NO$	
Formula weight	344.22	
Temperature	123(2) K	
Wavelength	0.71073 Å	
Crystal system	Monoclinic	
Space group	$P2_1/c$	
Unit cell dimensions	a = 13.9329(3) Å	α= 90°.
	b = 8.3923(2) Å	β= 94.0190(10)°.
	c = 12.8666(3) Å	γ = 90°.
Volume	1500.78(6) $Å^3$	
Z	4	
Density (calculated)	1.523 Mg/m^3	
Absorption coefficient	2.764 mm^{-1}	
F(000)	704	
Crystal size	0.30 x 0.20 x 0.10 mm^3	
Theta range for data collection	2.93 to 25.25°.	
Index ranges	-16<=h<=16, -8<=k<=10, -15<=l<=15	
Reflections collected	8720	
Independent reflections	2712 [R(int) = 0.0258]	
Completeness to theta = 25.25°	99.8 %	
Absorption correction	Semi-empirical from equivalents	
Max. and min. transmission	0.7696 and 0.4911	
Refinement method	Full-matrix least-squares on F^2	
Data / restraints / parameters	2712 / 2 / 178	
Goodness-of-fit on F^2	1.049	
Final R indices [$I>2\sigma(I)$]	R1 = 0.0227, wR2 = 0.0524	
R indices (all data)	R1 = 0.0292, wR2 = 0.0542	
Largest diff. peak and hole	0.313 and -0.244 e.$Å^{-3}$	

Restraints: DFIX (s = 0.02)

Table S7. Crystal data and structure refinement for (5).

Empirical formula	$C_{13}H_{20}BrF_2N$	
Formula weight	308.21	
Temperature	123(2) K	
Wavelength	0.71073 Å	
Crystal system	Orthorhombic	
Space group	$Pna2_1$	
Unit cell dimensions	a = 13.4970(2) Å	α= 90°.
	b = 13.2580(2) Å	β= 90°.
	c = 7.79800(10) Å	γ = 90°.
Volume	1395.40(3) $Å^3$	
Z	4	
Density (calculated)	1.467 Mg/m^3	
Absorption coefficient	2.948 mm^{-1}	
F(000)	632	
Crystal size	0.33 x 0.21 x 0.19 mm^3	
Theta range for data collection	3.02 to 25.24°.	
Index ranges	-15<=h<=16, -15<=k<=14, -9<=l<=9	
Reflections collected	17465	
Independent reflections	2530 [R(int) = 0.0387]	
Completeness to theta = 25.24°	99.9 %	
Absorption correction	Semi-empirical from equivalents	
Max. and min. transmission	0.6043 and 0.4429	
Refinement method	Full-matrix least-squares on F^2	
Data / restraints / parameters	2530 / 1 / 154	
Goodness-of-fit on F^2	1.067	
Final R indices [I>2σ(I)]	R1 = 0.0217, wR2 = 0.0490	
R indices (all data)	R1 = 0.0242, wR2 = 0.0501	
Absolute structure parameter	0.064(9)	
Largest diff. peak and hole	0.175 and -0.439 e.$Å^{-3}$	

Restraint: floating origin

Table S8. Crystal data and structure refinement for (6).

Empirical formula	$C_{13}H_{17}F_5IN$	
Formula weight	409.18	
Temperature	123(2) K	
Wavelength	0.71073 Å	
Crystal system	Monoclinic	
Space group	$P2_1/n$	
Unit cell dimensions	a = 8.01450(10) Å	α= 90°.
	b = 13.8741(2) Å	β= 95.0150(10)°.
	c = 13.5995(2) Å	γ = 90°.
Volume	1506.39(4) Å^3	
Z	4	
Density (calculated)	1.804 Mg/m^3	
Absorption coefficient	2.169 mm^{-1}	
F(000)	800	
Crystal size	0.50 x 0.25 x 0.19 mm^3	
Theta range for data collection	2.10 to 25.02°.	
Index ranges	-9<=h<=8, -16<=k<=16, -16<=l<=16	
Reflections collected	8756	
Independent reflections	2656 [R(int) = 0.0194]	
Completeness to theta = 25.02°	99.9 %	
Absorption correction	Semi-empirical from equivalents	
Max. and min. transmission	0.6834 and 0.4102	
Refinement method	Full-matrix least-squares on F^2	
Data / restraints / parameters	2656 / 0 / 181	
Goodness-of-fit on F^2	1.038	
Final R indices [I>2σ(I)]	R1 = 0.0189, wR2 = 0.0427	
R indices (all data)	R1 = 0.0210, wR2 = 0.0435	
Largest diff. peak and hole	0.476 and -0.387 e.Å^{-3}	

Table S9. Crystal data and structure refinement for (7).

Empirical formula	$C_{14}H_{11}BrF_5N$	
Formula weight	368.15	
Temperature	123(2) K	
Wavelength	0.71073 Å	
Crystal system	Monoclinic	
Space group	$P2_1/c$	
Unit cell dimensions	a = 5.7503(2) Å	α= 90°.
	b = 25.0706(9) Å	β= 94.871(2)°.
	c = 9.5048(4) Å	γ = 90°.
Volume	1365.30(9) $Å^3$	
Z	4	
Density (calculated)	1.791 Mg/m^3	
Absorption coefficient	3.060 mm^{-1}	
F(000)	728	
Crystal size	0.22 x 0.14 x 0.12 mm^3	
Theta range for data collection	2.70 to 25.02°.	
Index ranges	-5<=h<=6, -29<=k<=29, -11<=l<=11	
Reflections collected	14953	
Independent reflections	2389 [R(int) = 0.0833]	
Completeness to theta = 25.02°	99.1 %	
Absorption correction	Semi-empirical from equivalents	
Max. and min. transmission	0.7103 and 0.5525	
Refinement method	Full-matrix least-squares on F^2	
Data / restraints / parameters	2389 / 0 / 190	
Goodness-of-fit on F^2	1.077	
Final R indices [I>2σ(I)]	R1 = 0.0884, wR2 = 0.2410	
R indices (all data)	R1 = 0.1119, wR2 = 0.2581	
Largest diff. peak and hole	4.386 and -0.835 e.$Å^{-3}$	

Table S10. Crystal data and structure refinement for (8).

Empirical formula	$C_{16}H_{16}BrF_4N$	
Formula weight	378.21	
Temperature	123(2) K	
Wavelength	0.71073 Å	
Crystal system	Monoclinic	
Space group	$P2_1/n$	
Unit cell dimensions	a = 8.0793(2) Å	α= 90°.
	b = 16.9865(3) Å	β= 93.1410(10)°.
	c = 11.3472(2) Å	γ = 90°.
Volume	1554.94(5) Å^3	
Z	4	
Density (calculated)	1.616 Mg/m^3	
Absorption coefficient	2.680 mm^{-1}	
F(000)	760	
Crystal size	0.40 x 0.19 x 0.06 mm^3	
Theta range for data collection	2.40 to 25.02°.	
Index ranges	-9<=h<=9, -20<=k<=20, -12<=l<=13	
Reflections collected	9119	
Independent reflections	2750 [R(int) = 0.0349]	
Completeness to theta = 25.02°	99.9 %	
Absorption correction	Semi-empirical from equivalents	
Max. and min. transmission	0.8557 and 0.4136	
Refinement method	Full-matrix least-squares on F^2	
Data / restraints / parameters	2750 / 0 / 199	
Goodness-of-fit on F^2	1.101	
Final R indices [I>2σ(I)]	R1 = 0.0270, wR2 = 0.0601	
R indices (all data)	R1 = 0.0378, wR2 = 0.0625	
Largest diff. peak and hole	0.280 and -0.306 e.Å^{-3}	

Table S11. Crystal data and structure refinement for (9).

Empirical formula	$C_{16}H_{17}BrF_3N$	
Formula weight	360.22	
Temperature	123(2) K	
Wavelength	0.71073 Å	
Crystal system	Orthorhombic	
Space group	*Pna*2_1	
Unit cell dimensions	a = 11.7838(2) Å	α= 90°.
	b = 20.3034(4) Å	β= 90°.
	c = 6.37720(10) Å	γ = 90°.
Volume	1525.75(5) $Å^3$	
Z	4	
Density (calculated)	1.568 Mg/m^3	
Absorption coefficient	2.718 mm^{-1}	
F(000)	728	
Crystal size	0.18 x 0.16 x 0.13 mm^3	
Theta range for data collection	2.65 to 25.25°.	
Index ranges	-13<=h<=14, -23<=k<=24, -7<=l<=6	
Reflections collected	18750	
Independent reflections	2693 [R(int) = 0.0466]	
Completeness to theta = 25.25°	99.8 %	
Absorption correction	Semi-empirical from equivalents	
Max. and min. transmission	0.7189 and 0.6404	
Refinement method	Full-matrix least-squares on F^2	
Data / restraints / parameters	2693 / 1 / 190	
Goodness-of-fit on F^2	1.056	
Final R indices [I>2σ(I)]	R1 = 0.0235, wR2 = 0.0491	
R indices (all data)	R1 = 0.0274, wR2 = 0.0505	
Absolute structure parameter	0.085(8)	
Largest diff. peak and hole	0.198 and -0.192 e.$Å^{-3}$	

Restraint: floating origin

Table S12. Crystal data and structure refinement for (10).

Empirical formula	$C_{26}H_{34}Cl_2F_6N_4O$	
Formula weight	603.47	
Temperature	123(2) K	
Wavelength	0.71073 Å	
Crystal system	Monoclinic	
Space group	*C*2/*c*	
Unit cell dimensions	a = 10.5705(2) Å	α= 90°.
	b = 9.9886(3) Å	β= 95.7420(10)°.
	c = 26.3808(5) Å	γ = 90°.
Volume	2771.43(11) $Å^3$	
Z	4	
Density (calculated)	1.446 Mg/m^3	
Absorption coefficient	0.303 mm^{-1}	
F(000)	1256	
Crystal size	0.35 x 0.20 x 0.10 mm^3	
Theta range for data collection	2.81 to 25.02°.	
Index ranges	-12<=h<=12, -11<=k<=11, -31<=l<=31	
Reflections collected	4647	
Independent reflections	2450 [R(int) = 0.0262]	
Completeness to theta = 25.02°	100.0 %	
Absorption correction	None	
Refinement method	Full-matrix least-squares on F^2	
Data / restraints / parameters	2450 / 1 / 180	
Goodness-of-fit on F^2	1.049	
Final R indices [I>2σ(I)]	R1 = 0.0359, wR2 = 0.0811	
R indices (all data)	R1 = 0.0439, wR2 = 0.0849	
Largest diff. peak and hole	0.233 and -0.203 e.$Å^{-3}$	

Restraint: DFIX (s = 0.02)

Table S13. Crystal data and structure refinement for (11).

Empirical formula	$C_{12}H_8BrF_5N_2$	
Formula weight	355.11	
Temperature	123(2) K	
Wavelength	0.71073 Å	
Crystal system	Trigonal	
Space group	$P3_1$	
Unit cell dimensions	a = 8.72990(10) Å	α= 90°.
	b = 8.72990(10) Å	β= 90°.
	c = 14.3094(3) Å	γ = 120°.
Volume	944.43(3) $Å^3$	
Z	3	
Density (calculated)	1.873 Mg/m^3	
Absorption coefficient	3.316 mm^{-1}	
F(000)	522	
Crystal size	0.50 x 0.48 x 0.42 mm^3	
Theta range for data collection	3.92 to 25.21°.	
Index ranges	-10<=h<=10, -10<=k<=9, -16<=l<=17	
Reflections collected	5427	
Independent reflections	2170 [R(int) = 0.0200]	
Completeness to theta = 25.21°	99.6 %	
Absorption correction	Semi-empirical from equivalents	
Max. and min. transmission	0.3364 and 0.2879	
Refinement method	Full-matrix least-squares on F^2	
Data / restraints / parameters	2170 / 3 / 187	
Goodness-of-fit on F^2	1.087	
Final R indices [I>2σ(I)]	R1 = 0.0189, wR2 = 0.0381	
R indices (all data)	R1 = 0.0199, wR2 = 0.0384	
Absolute structure parameter	0.035(6)	
Largest diff. peak and hole	0.197 and -0.319 e.$Å^{-3}$	

Restraints: floating origin, DFIX (s = 0.02)

Table S14. Crystal data and structure refinement for (12).

Empirical formula	$C_{12}H_8BrF_5N_2$	
Formula weight	355.11	
Temperature	123(2) K	
Wavelength	0.71073 Å	
Crystal system	Monoclinic	
Space group	$P2_1/n$	
Unit cell dimensions	a = 11.4302(2) Å	α= 90°.
	b = 9.47480(10) Å	β= 98.8540(10)°.
	c = 23.7921(5) Å	γ = 90°.
Volume	2545.95(7) $Å^3$	
Z	8	
Density (calculated)	1.853 Mg/m^3	
Absorption coefficient	3.280 mm^{-1}	
F(000)	1392	
Crystal size	0.52 x 0.09 x 0.07 mm^3	
Theta range for data collection	2.76 to 25.25°.	
Index ranges	-13<=h<=13, -11<=k<=11, -28<=l<=28	
Reflections collected	8878	
Independent reflections	4612 [R(int) = 0.0241]	
Completeness to theta = 25.25°	99.8 %	
Absorption correction	Semi-empirical from equivalents	
Max. and min. transmission	0.8029 and 0.2803	
Refinement method	Full-matrix least-squares on F^2	
Data / restraints / parameters	4612 / 4 / 373	
Goodness-of-fit on F^2	1.034	
Final R indices [I>2σ(I)]	R1 = 0.0271, wR2 = 0.0550	
R indices (all data)	R1 = 0.0386, wR2 = 0.0587	
Largest diff. peak and hole	0.278 and -0.281 e.$Å^{-3}$	

Restraints: DFIX (s = 0.02)

Table S15. Crystal data and structure refinement for (13).

Empirical formula	$C_{17}H_{12}BrF_5N_2O$	
Formula weight	435.20	
Temperature	123(2) K	
Wavelength	0.71073 Å	
Crystal system	Triclinic	
Space group	*P*-1	
Unit cell dimensions	a = 13.6652(3) Å	α= 77.611(2)°.
	b = 14.6348(3) Å	β= 73.769(2)°.
	c = 18.0643(3) Å	γ = 75.674(2)°.
Volume	3319.49(11) $Å^3$	
Z	8	
Density (calculated)	1.742 Mg/m^3	
Absorption coefficient	2.538 mm^{-1}	
F(000)	1728	
Crystal size	0.15 x 0.13 x 0.10 mm^3	
Theta range for data collection	2.52 to 25.25°.	
Index ranges	-16<=h<=15, -17<=k<=17, -21<=l<=21	
Reflections collected	20164	
Independent reflections	12016 [R(int) = 0.0347]	
Completeness to theta = 25.25°	99.8 %	
Absorption correction	Semi-empirical from equivalents	
Max. and min. transmission	0.7854 and 0.7020	
Refinement method	Full-matrix least-squares on F^2	
Data / restraints / parameters	12016 / 12 / 961	
Goodness-of-fit on F^2	1.039	
Final R indices [I>2σ(I)]	R1 = 0.0433, wR2 = 0.0806	
R indices (all data)	R1 = 0.0734, wR2 = 0.0909	
Largest diff. peak and hole	0.503 and -0.484 e.$Å^{-3}$	

Restraints: DFIX (s = 0.02)

Table S16. Crystal data and structure refinement for (14).

Empirical formula	$C_{11}H_8BrF_5N_2$	
Formula weight	343.10	
Temperature	123(2) K	
Wavelength	0.71073 Å	
Crystal system	Monoclinic	
Space group	$P2_1/n$	
Unit cell dimensions	a = 10.1966(2) Å	α= 90°.
	b = 11.8976(2) Å	β= 101.2530(10)°.
	c = 10.2358(2) Å	γ = 90°.
Volume	1217.88(4) $Å^3$	
Z	4	
Density (calculated)	1.871 Mg/m^3	
Absorption coefficient	3.425 mm^{-1}	
F(000)	672	
Crystal size	0.30 x 0.25 x 0.23 mm^3	
Theta range for data collection	3.42 to 25.02°.	
Index ranges	-12<=h<=12, -14<=k<=13, -12<=l<=12	
Reflections collected	7035	
Independent reflections	2137 [R(int) = 0.0232]	
Completeness to theta = 25.02°	99.6 %	
Absorption correction	Semi-empirical from equivalents	
Max. and min. transmission	0.5063 and 0.4264	
Refinement method	Full-matrix least-squares on F^2	
Data / restraints / parameters	2137 / 0 / 173	
Goodness-of-fit on F^2	1.057	
Final R indices [I>2σ(I)]	R1 = 0.0217, wR2 = 0.0517	
R indices (all data)	R1 = 0.0261, wR2 = 0.0530	
Largest diff. peak and hole	0.251 and -0.254 e.$Å^{-3}$	

Table S17. Crystal data and structure refinement for (15).

Empirical formula	$C_{21}H_{11}BrF_{10}N_2O$	
Formula weight	577.23	
Temperature	123(2) K	
Wavelength	0.71073 Å	
Crystal system	Monoclinic	
Space group	$P2_1/c$	
Unit cell dimensions	a = 12.4829(3) Å	α= 90°.
	b = 16.1450(4) Å	β= 91.5290(10)°.
	c = 10.3729(2) Å	γ = 90°.
Volume	2089.77(8) $Å^3$	
Z	4	
Density (calculated)	1.835 Mg/m^3	
Absorption coefficient	2.074 mm^{-1}	
F(000)	1136	
Crystal size	0.25 x 0.18 x 0.14 mm^3	
Theta range for data collection	2.33 to 25.02°.	
Index ranges	-13<=h<=14, -18<=k<=19, -12<=l<=12	
Reflections collected	26660	
Independent reflections	3690 [R(int) = 0.0623]	
Completeness to theta = 25.02°	99.9 %	
Absorption correction	Semi-empirical from equivalents	
Max. and min. transmission	0.7600 and 0.6251	
Refinement method	Full-matrix least-squares on F^2	
Data / restraints / parameters	3690 / 2 / 328	
Goodness-of-fit on F^2	1.060	
Final R indices [I>2σ(I)]	R1 = 0.0614, wR2 = 0.1701	
R indices (all data)	R1 = 0.0778, wR2 = 0.1818	
Largest diff. peak and hole	0.691 and -0.954 e.$Å^{-3}$	

Restraints: DFIX (s = 0.02)

Table S18. Crystal data and structure refinement for (16A).

Empirical formula	$C_{13}H_{16}BrF_5N_2$	
Formula weight	375.19	
Temperature	123(2) K	
Wavelength	0.71073 Å	
Crystal system	Monoclinic	
Space group	$P2_1/n$	
Unit cell dimensions	a = 8.9614(3) Å	α= 90°.
	b = 18.5641(7) Å	β= 94.771(2)°.
	c = 8.8560(3) Å	γ = 90°.
Volume	1468.18(9) $Å^3$	
Z	4	
Density (calculated)	1.697 Mg/m^3	
Absorption coefficient	2.849 mm^{-1}	
F(000)	752	
Crystal size	0.12 x 0.07 x 0.04 mm^3	
Theta range for data collection	2.53 to 25.25°.	
Index ranges	-10<=h<=10, -22<=k<=22, -10<=l<=10	
Reflections collected	5195	
Independent reflections	2653 [R(int) = 0.0323]	
Completeness to theta = 25.25°	99.7 %	
Absorption correction	Semi-empirical from equivalents	
Max. and min. transmission	0.8946 and 0.7262	
Refinement method	Full-matrix least-squares on F^2	
Data / restraints / parameters	2653 / 0 / 190	
Goodness-of-fit on F^2	1.192	
Final R indices [I>2σ(I)]	R1 = 0.0583, wR2 = 0.1277	
R indices (all data)	R1 = 0.0838, wR2 = 0.1357	
Largest diff. peak and hole	0.684 and -0.385 e.$Å^{-3}$	

Table S19. Crystal data and structure refinement for (16B).

Empirical formula	$C_{13}H_{16}BrF_5N_2$	
Formula weight	375.19	
Temperature	123(2) K	
Wavelength	0.71073 Å	
Crystal system	Monoclinic	
Space group	$P2_1/n$	
Unit cell dimensions	a = 8.8555(2) Å	α= 90°.
	b = 18.5382(5) Å	β= 94.773(2)°.
	c = 8.9536(2) Å	γ = 90°.
Volume	1464.77(6) $Å^3$	
Z	4	
Density (calculated)	1.701 Mg/m^3	
Absorption coefficient	2.855 mm^{-1}	
F(000)	752	
Crystal size	0.34 x 0.07 x 0.05 mm^3	
Theta range for data collection	2.53 to 25.02°.	
Index ranges	-10<=h<=10, -22<=k<=22, -10<=l<=10	
Reflections collected	8527	
Independent reflections	2579 [R(int) = 0.0348]	
Completeness to theta = 25.02°	99.4 %	
Absorption correction	Semi-empirical from equivalents	
Max. and min. transmission	0.8704 and 0.4435	
Refinement method	Full-matrix least-squares on F^2	
Data / restraints / parameters	2579 / 0 / 190	
Goodness-of-fit on F^2	1.242	
Final R indices [I>2σ(I)]	R1 = 0.0504, wR2 = 0.1145	
R indices (all data)	R1 = 0.0637, wR2 = 0.1181	
Largest diff. peak and hole	0.403 and -0.387 e.$Å^{-3}$	

Table S20. Crystal data and structure refinement for (17).

Empirical formula	$C_{11}H_{13}BrF_5NO$	
Formula weight	350.13	
Temperature	123(2) K	
Wavelength	0.71073 Å	
Crystal system	Monoclinic	
Space group	$P2_1/n$	
Unit cell dimensions	a = 6.86600(10) Å	α= 90°.
	b = 14.2952(3) Å	β= 94.4170(10)°.
	c = 26.3661(5) Å	γ = 90°.
Volume	2580.17(8) $Å^3$	
Z	8	
Density (calculated)	1.803 Mg/m^3	
Absorption coefficient	3.238 mm^{-1}	
F(000)	1392	
Crystal size	0.38 x 0.30 x 0.24 mm^3	
Theta range for data collection	1.55 to 25.25°.	
Index ranges	-8<=h<=8, -17<=k<=17, -31<=l<=31	
Reflections collected	9105	
Independent reflections	4666 [R(int) = 0.0175]	
Completeness to theta = 25.25°	100.0 %	
Absorption correction	Semi-empirical from equivalents	
Max. and min. transmission	0.5104 and 0.3725	
Refinement method	Full-matrix least-squares on F^2	
Data / restraints / parameters	4666 / 2 / 367	
Goodness-of-fit on F^2	1.031	
Final R indices [I>2σ(I)]	R1 = 0.0245, wR2 = 0.0540	
R indices (all data)	R1 = 0.0311, wR2 = 0.0568	
Largest diff. peak and hole	0.238 and -0.341 e.$Å^{-3}$	

Restraints: DFIX (s = 0.02)

Table S21. Crystal data and structure refinement for (18A).

Empirical formula	$C_{22}H_{26}BrClF_{10}N_2OS$	
Formula weight	671.87	
Temperature	123(2) K	
Wavelength	0.71073 Å	
Crystal system	Triclinic	
Space group	*P*-1	
Unit cell dimensions	a = 6.9241(2) Å	α= 111.6180(10)°.
	b = 12.7624(3) Å	β= 90.966(2)°.
	c = 16.7270(4) Å	γ = 93.449(2)°.
Volume	1370.46(6) Å^3	
Z	2	
Density (calculated)	1.628 Mg/m^3	
Absorption coefficient	1.761 mm^{-1}	
F(000)	676	
Crystal size	0.44 x 0.40 x 0.27 mm^3	
Theta range for data collection	2.62 to 25.25°.	
Index ranges	-8<=h<=8, -15<=k<=15, -20<=l<=17	
Reflections collected	8300	
Independent reflections	4939 [R(int) = 0.0181]	
Completeness to theta = 25.25°	99.6 %	
Absorption correction	Semi-empirical from equivalents	
Max. and min. transmission	0.6478 and 0.5113	
Refinement method	Full-matrix least-squares on F^2	
Data / restraints / parameters	4939 / 37 / 343	
Goodness-of-fit on F^2	1.037	
Final R indices [I>2sigma(I)]	R1 = 0.0347, wR2 = 0.0851	
R indices (all data)	R1 = 0.0436, wR2 = 0.0897	
Largest diff. peak and hole	0.403 and -0.602 e.Å^{-3}	

Restraints: SIMU (36, s = 0.01), DELU (1, s = 0.01); EXYZ and EADP constraints utilized in halide disorder

Table S22. Crystal data and structure refinement for (18B).

Empirical formula	$C_{21}H_{24}Br_2F_{10}N_2O$	
Formula weight	670.24	
Temperature	123(2) K	
Wavelength	0.71073 Å	
Crystal system	Monoclinic	
Space group	$P2_1/c$	
Unit cell dimensions	a = 13.2107(3) Å	α= 90°.
	b = 7.19550(10) Å	β= 91.1490(10)°.
	c = 25.9512(6) Å	γ = 90°.
Volume	2466.36(9) $Å^3$	
Z	4	
Density (calculated)	1.805 Mg/m^3	
Absorption coefficient	3.380 mm^{-1}	
F(000)	1328	
Crystal size	0.22 x 0.18 x 0.04 mm^3	
Theta range for data collection	1.57 to 25.25°.	
Index ranges	-15<=h<=15, -8<=k<=8, -31<=l<=31	
Reflections collected	15646	
Independent reflections	4457 [R(int) = 0.0523]	
Completeness to theta = 25.25°	99.9 %	
Absorption correction	Semi-empirical from equivalents	
Max. and min. transmission	0.8766 and 0.5234	
Refinement method	Full-matrix least-squares on F^2	
Data / restraints / parameters	4457 / 1 / 328	
Goodness-of-fit on F^2	1.059	
Final R indices [I>2σ(I)]	R1 = 0.0336, wR2 = 0.0608	
R indices (all data)	R1 = 0.0498, wR2 = 0.0650	
Largest diff. peak and hole	0.354 and -0.363 e.$Å^{-3}$	

Restraint: DFIX (s = 0.02)

Table S23. Crystal data and structure refinement for (18C).

Empirical formula	$C_{22}H_{26}Br_2F_{10}N_2OS$	
Formula weight	716.33	
Temperature	123(2) K	
Wavelength	0.71073 Å	
Crystal system	Triclinic	
Space group	*P*-1	
Unit cell dimensions	a = 7.1359(2) Å	α= 111.3640(10)°.
	b = 12.6440(3) Å	β= 91.7140(10)°.
	c = 16.7753(4) Å	γ = 92.9300(10)°.
Volume	1405.81(6) Å^3	
Z	2	
Density (calculated)	1.692 Mg/m^3	
Absorption coefficient	3.043 mm^{-1}	
F(000)	712	
Crystal size	0.20 x 0.18 x 0.13 mm^3	
Theta range for data collection	2.86 to 25.25°.	
Index ranges	-8<=h<=8, -13<=k<=15, -20<=l<=19	
Reflections collected	8524	
Independent reflections	5077 [R(int) = 0.0190]	
Completeness to theta = 25.25°	99.6 %	
Absorption correction	Semi-empirical from equivalents	
Max. and min. transmission	0.6931 and 0.5813	
Refinement method	Full-matrix least-squares on F^2	
Data / restraints / parameters	5077 / 30 / 343	
Goodness-of-fit on F^2	1.055	
Final R indices [I>2sigma(I)]	R1 = 0.0530, wR2 = 0.1467	
R indices (all data)	R1 = 0.0641, wR2 = 0.1551	
Largest diff. peak and hole	1.924 and -1.087 e.Å^{-3}	

Restraints: SADI (6, s = 0,02), ISOR (24, s = 0.02); EADP constraint utilized in solvent disorder

Table S24. Crystal data and structure refinement for (19).

Empirical formula	$C_{51.80}H_{57.40}Br_4Cl_9F_{20}N_4O_{2.80}S_4$	
Formula weight	1927.75	
Temperature	123(2) K	
Wavelength	0.71073 Å	
Crystal system	Monoclinic	
Space group	$P2_1/c$	
Unit cell dimensions	a = 17.8941(5) Å	α= 90°.
	b = 10.1069(3) Å	β= 101.532(2)°.
	c = 10.1222(3) Å	γ = 90°.
Volume	1793.68(9) $Å^3$	
Z	1	
Density (calculated)	1.785 Mg/m^3	
Absorption coefficient	2.791 mm^{-1}	
F(000)	956	
Crystal size	0.30 x 0.20 x 0.03 mm^3	
Theta range for data collection	2.88 to 25.02°.	
Index ranges	-21<=h<=20, -8<=k<=12, -12<=l<=12	
Reflections collected	18769	
Independent reflections	3138 [R(int) = 0.0558]	
Completeness to theta = 25.02°	99.7 %	
Absorption correction	Semi-empirical from equivalents	
Max. and min. transmission	0.9209 and 0.4881	
Refinement method	Full-matrix least-squares on F^2	
Data / restraints / parameters	3138 / 2 / 244	
Goodness-of-fit on F^2	1.034	
Final R indices [I>2σ(I)]	R1 = 0.0377, wR2 = 0.0705	
R indices (all data)	R1 = 0.0537, wR2 = 0.0757	
Largest diff. peak and hole	0.526 and -0.363 e.$Å^{-3}$	

Restraints: DFIX (s = 0.02); EADP constraint utilized in solvent disorder

Table S25. Crystal data and structure refinement for (20).

Empirical formula	$C_{13}H_{20}ClF_5N_2O$	
Formula weight	350.76	
Temperature	123(2) K	
Wavelength	0.71073 Å	
Crystal system	Monoclinic	
Space group	$P2_1/c$	
Unit cell dimensions	a = 12.7577(2) Å	α= 90°.
	b = 8.8912(2) Å	β= 112.3260(10)°.
	c = 15.3866(3) Å	γ = 90°.
Volume	1614.49(5) $Å^3$	
Z	4	
Density (calculated)	1.443 Mg/m^3	
Absorption coefficient	0.290 mm^{-1}	
F(000)	728	
Crystal size	0.30 x 0.27 x 0.20 mm^3	
Theta range for data collection	2.70 to 25.25°.	
Index ranges	-15<=h<=15, -10<=k<=10, -18<=l<=18	
Reflections collected	5629	
Independent reflections	2931 [R(int) = 0.0159]	
Completeness to theta = 25.25°	99.9 %	
Absorption correction	Semi-empirical from equivalents	
Max. and min. transmission	0.9443 and 0.9181	
Refinement method	Full-matrix least-squares on F^2	
Data / restraints / parameters	2931 / 2 / 205	
Goodness-of-fit on F^2	1.045	
Final R indices [I>2σ(I)]	R1 = 0.0311, wR2 = 0.0721	
R indices (all data)	R1 = 0.0365, wR2 = 0.0750	
Largest diff. peak and hole	0.236 and -0.203 e.$Å^{-3}$	

Restraints: DFIX (s = 0.02)

Table S26. Crystal data and structure refinement for (21).

Empirical formula	$C_{12}H_7F_5IN_3O$	
Formula weight	431.11	
Temperature	123(2) K	
Wavelength	0.71073 Å	
Crystal system	Orthorhombic	
Space group	$P2_12_12_1$	
Unit cell dimensions	a = 9.7161(3) Å	α= 90°.
	b = 11.9730(3) Å	β= 90°.
	c = 12.5893(3) Å	γ = 90°.
Volume	1464.52(7) $Å^3$	
Z	4	
Density (calculated)	1.955 Mg/m^3	
Absorption coefficient	2.245 mm^{-1}	
F(000)	824	
Crystal size	0.29 x 0.11 x 0.10 mm^3	
Theta range for data collection	2.65 to 25.24°.	
Index ranges	-11<=h<=11, -14<=k<=13, -14<=l<=15	
Reflections collected	18585	
Independent reflections	2647 [R(int) = 0.0391]	
Completeness to theta = 25.24°	99.9 %	
Absorption correction	Semi-empirical from equivalents	
Max. and min. transmission	0.8066 and 0.5621	
Refinement method	Full-matrix least-squares on F^2	
Data / restraints / parameters	2647 / 5 / 211	
Goodness-of-fit on F^2	1.051	
Final R indices [I>2σ(I)]	R1 = 0.0196, wR2 = 0.0479	
R indices (all data)	R1 = 0.0204, wR2 = 0.0482	
Absolute structure parameter	0.075(19)	
Largest diff. peak and hole	0.298 and -0.365 e.$Å^{-3}$	

Restraints: DFIX (s = 0.02)

Determination of the anion position relative to the electron-deficient arene

In order to compare the positions of the anions in respect to the aromatic moiety a generalized Cartesian system was used. The arene was placed in the x-z-plane centered in the origin (see figure b). For each anion the distance of the anion in respect to the plane (y-coordinates) was determined from the obtained crystal structures. By measuring the distances between the anions and C_2, C_4 and C_6 the x- and z-coordinate were calculated. The obtained positions are listed in the following table.

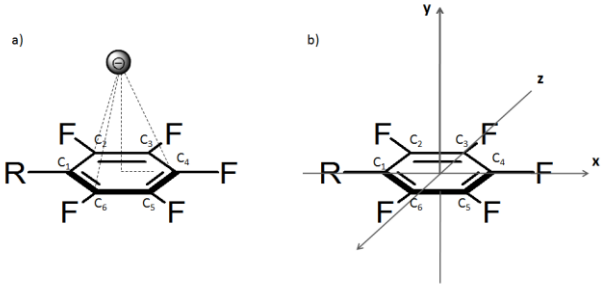

Table S27. Determinded position of the anion relative to the fluorinated arene.

Anion	x	y	z
Cl12	-2.057	3.294	1.997
Cl13	1.980	3.247	-1.262
Cl14	-0.037	3.370	0.468
Cl15	-0.120	3.312	-1.035
Cl16	-3.276	-0.466	-3.704
Cl17	-0.010	3.586	0.278
Cl18	-3.065	2.868	-2.461
Cl19	-2.042	3.012	-2.603
Br20	0.736	3.453	1.923
Br21	1.214	3.391	-1.452
Br22	-2.008	0.741	-4.812
Br22	-0.753	3.579	-0.389
Br24	-4.739	-0.578	-3.336
Br25	-0.734	3.420	-1.803
Br26	0.021	3.517	0.569
Br27	-5.021	-0.642	-3.822
Br28	0.261	3.467	-0.928
Br29	0.444	3.808	1.314
Br30	0.038	3.569	-0.119
Br31	-0.386	3.765	-0.547
Br32	-0.478	4.081	0.808
Br33	-0.500	3.756	2.495
Br34	0.261	3.470	-1.999
Br35	-0.622	3.644	2.479
Br36	-0.336	3.463	1.431
Br37	-4.247	-0.251	3.536
Br38	0.532	3.520	-1.097
Br39	0.777	3.409	0.071

Br40	-1.405	5.010	-3.042
Br41	-3.099	0.002	4.184
Br42	-3.120	-1.503	3.688
Br43	-3.423	-0.865	-3.602
Br44	-0.479	3.471	3.299
Br45	0.091	3.547	0.876
Br46	0.029	3.614	2.983
Br47	0.737	3.394	-0.772
Br48	-0.080	3.749	0.472
Br49	0.181	3.290	2.192
Br50	0.276	3.492	0.903
Br51	0.553	3.380	-1.373
Br52	0.651	3.401	0.042
Br53	0.403	3.378	1.791
Br54	0.835	3.417	-0.536
Br55	0.338	3.428	-0.050
Br56	3.365	-1.117	-3.434
Br57	5.004	0.302	-0.574
Br58	-1.039	4.417	-1.292
Br59	3.002	3.454	1.824
Br60	0.064	3.461	-0.287
Br61	0.306	3.424	-0.589
Br62	0.381	3.498	-2.060
Br63	0.424	3.808	1.365
Br64	0.266	3.484	-0.885
Br65	0.692	3.585	-1.362
Br66	-0.706	3.587	1.422
Br67	0.814	3.367	0.340
Br68	-1.316	3.727	2.031
Br69	-1.206	-3.276	-2.141
Br70	0.000	3.213	2.097
Br71	-0.036	3.657	0.190
Br72	0.150	3.653	-0.346
Br73	-4.077	0.961	3.554
Br74	-3.162	2.965	2.637
Br75	-2.447	3.156	2.555
Br76	0.387	3.595	0.561
Br77	0.209	3.826	0.546
I78	0.183	3.839	-0.123
I79	0.135	3.906	0.537
I80	0.157	4.180	-1.189
I81	0.399	3.927	1.722
I82	0.457	3.629	-0.171
I83	0.234	3.772	0.003
I84	-0.603	3.830	-2.151
I85	-0.344	3.720	-0.861

I86	-1.523	3.552	-0.123
I87	-0.208	-3.571	0.681
I88	1.494	3.595	0.346
I89	-0.203	3.617	-0.247
I90	0.480	4.302	-1.545
I91	0.473	3.490	2.408
I92	-0.555	-3.887	-0.624
I93	0.990	3.721	1.543
I94	1.972	3.413	-1.220
I95	0.261	3.730	0.536
Co96	0.752	3.933	0.611
Co97	0.855	3.897	-1.388
Co98	0.447	4.374	1.363
Co99	0.164	3.020	-4.026
Co100	1.082	3.831	1.559
Co101	-0.422	3.025	-0.757
Co102	1.367	3.555	-0.649
Co103	2.183	3.981	0.156
Co104	-0.498	3.825	-2.117
Co105	-0.494	4.102	-1.758
Co106	-0.011	3.892	0.522
Co107	0.451	3.852	-0.027
Co108	0.713	3.703	-0.065
Co109	0.147	4.010	0.481
Co110	-0.162	4.028	0.708
Co111	-1.705	-4.954	0.910
Co112	0.533	3.816	2.220

III. References

[1] M. Giese**,** M. Albrecht, K. Wiemer, A. Valkonen, K. Rissanen, *New J. Chem.* **2012**, 1368-1372.
[2] M. Albrecht, M. Müller, O. Mergel, K. Rissanen, A. Valkonen, *Chem. Eur. J.* **2010**, *16*, 5062.
[3] M. Giese, M. Albrecht, C. Plum, D. Hintzen, A. Valkonen, K. Rissanen, *Supramol. Chem.* **2012**, *24*, 755-761.
[4] M. Albrecht, M. Müller, A. Valkonen, K. Rissanen, *Cryst. Eng. Commun.* **2010**, *12*, 3698.
[5] M. Giese, M. Albrecht, C. Bannwarth, G. Raabe, A. Valkonen, K. Rissanen, *Chem. Commun.* **2011**, *47*, 8542.
[6] M. Giese, M. Albrecht, T. Krappitz, M. Peters, V. Gossen, G. Raabe, A. Valkonen, K. Rissanen, *Chem. Commun.* **2012**, *48*, 9983-9985.
[7] M. Giese, M. Albrecht, T. Repenko, J. Sackmann, A. Valkonen, K. Rissanen, *Eur. J. Org. Chem.* **2014**, *accepted for publication*. DOI: 10.1002/ejoc.201301336
[8] M. Giese**,** M. Albrecht, K. Wiemer, G. Kubik, A. Valkonen, K. Rissanen,*Eur. J. Inorg. Chem.* **2012**, 2995-2999.
[9] M. Giese, M. Albrecht, S. Steike, A. Ackermann, A. Valkonen, K. Rissanen, *Inorg. Chem.* **2013**, *52*, 7666-7672.
[10] M. Müller, M. Albrecht, V. Gossen, T. Peters, A. Hoffmann, G. Raabe, A. Valkonen, K. Rissanen, *Chem. Eur. J.* **2010**, *16*, 12446.
[11] M. Giese, M. Albrecht, A. Valkonen, K. Rissanen, *Eur. J. Org. Chem.* **2013**, 3247-3253.
[12] M. Müller, M. Albrecht, J. Sackmann, A. Hoffmann, F. Dierkes, A. Valkonen, K. Rissanen, *Dalton Trans.* **2010**, 39, 11329.
[13] M. Giese, M. Albrecht, C. Bohnen, T. Repenko, A. Valkonen, K. Rissanen, *Dalton Trans.* **2013**, *accepted for publication*.
[14] M. Giese, M. Albrecht, G. Ivanova, A. Valkonen, K. Rissanen, *Supramol. Chem.* **2012**, 1, 48-55.

[10] a) *COLLECT*, Bruker AXS, Inc., Madison, Wisconsin, USA, **2008**; b) Z. Otwinowski, W. Minor, in *Methods Enzymol.* **1997**, *276*, Macromolecular Crystallography, Part A, 307-326; c) M. C. Burla, R. Caliandro, M. Camalli, B. Carrozzini,G. L. Cascarano, L. De Caro, C. Giacovazzo, G. Polidori and R. Spagna, *J. Appl. Crystallogr.* **2005**, *38*, 381-388; d) L. J. Farrugia, *J. Appl. Crystallogr.* **1999**, *32*, 837-838; e) G. M. Sheldrick, *Acta Crystallogr., Sect. A: Found. Crystallogr.* **2008**, *64*, 112-122; f) G. M. Sheldrick, *SADABS*, University of Göttingen, Germany, **1996**.
